# X-ray-responsive dissolving microneedles mediate STING pathway activation to potentiate cutaneous melanoma radio-immunotherapy

**DOI:** 10.7150/thno.110841

**Published:** 2025-06-09

**Authors:** Wen Hu, Xiaohong Hong, Xinyu Zhang, Hongfan Chen, Xin Wen, Feng Lin, Jingwen Liu, Chenfenglin Yang, Binglin Cheng, Hanrui Zhu, Moting Zhang, Ruzhen Chen, Tingting Peng, Xinran Tang

**Affiliations:** 1 Department of Radiation Oncology, Nanfang Hospital, Southern Medical University, Guangzhou, 510515, China.; 2 State Key Laboratory of Bioactive Molecules and Druggability Assessment, Guangdong Basic Research Center of Excellence for Natural Bioactive Molecules and Discovery of Innovative Drugs, College of Pharmacy, Jinan University, Guangzhou, 511436, China.; 3 Southern Medical University, Guangzhou, 510515, China.; 4 Division of Hepatobiliopancreatic Surgery, Department of General Surgery, Nanfang Hospital, Southern Medical University, Guangzhou, 510515, China.

**Keywords:** dissolving microneedles, Mn-ZIF-8, radio-immunotherapy, melanoma, radiosensitization

## Abstract

**Background:** Radiotherapy (RT) often activates the cyclic GMP-AMP synthase (cGAS) stimulator of interferon response cGAMP interactor (STING) signaling pathway and induces systemic immunotherapy effects by triggering immunogenic cell death (ICD) in various solid tumors. However, RT-induced ICD usually falls short in eradicating distant tumors because of moderate anti-tumor immune responses.

**Methods:** In this study, Mn-ZIF-8 nanoparticles and microneedles were prepared, and their physical and chemical properties were characterized. Subsequently, *in vitro* experiments using B16 and A375 cutaneous melanoma cell lines were conducted to investigate the radiosensitivity characteristics of Mn-ZIF-8 and its mechanism for enhancing RT efficacy. Moreover, mouse models bearing primary and distant B16 cutaneous melanoma were established to clarify the immunomodulatory effect and antitumor efficacy of Mn-ZIF-8 microneedles when combined with RT and immunotherapy.

**Results:** A percutaneous delivery method based on soluble microneedles (MNs) with Mn^2+^-loaded, X-ray-responsive zeolite imidazolate frame-8 (ZIF-8) was designed. This microneedle-based drug delivery system, combined with RT, promoted the radiosensitivity of cutaneous melanoma and reinforces ICD by augmenting STING pathway activation. Furthermore, after X-ray irradiation, Mn-ZIF-8 MNs continuously released Mn^2+^ in the tumor to enhance cGAS-STING activation. This promoted dendritic cell maturation and antigen presentation, and potentiated a T cell mediated immune response. Thus, the local and systemic immune effects induced by RT were amplified when combined with immune checkpoint inhibitors.

**Conclusion:** The microneedle patches with X-ray-responsive, rapid dissolution and controlled release abilities have the potential to enhance the radioimmunotherapy efficacy for cutaneous melanoma.

## Introduction

Radiotherapy (RT) not only directly kills tumor cells through DNA damage caused by ionizing radiation, but also induces tumor regression beyond the irradiation field by activating an immune response, termed the "abscopal effect" of RT 1-3.This effect is mainly caused by immunogenic cell death (ICD), in which dendritic cells (DCs) recognize and present exposed tumor antigens to activated T cells to generate a systemic anti-tumor immune response, thereby diminishing metastatic cancer in non-irradiated areas 4, 5. However, the anti-tumor immune response induced by RT alone is usually insufficient to eliminate distant tumors 6, 7. The immunosuppressive tumor microenvironment (TME) often limits the therapeutic effects of RT 8-10. Therefore, it is critical to develop novel RT strategies to reshape the TME to overcome RT resistance and immunosuppression.

Immune checkpoint inhibitors (ICIs) can reinvigorate T cells in an immunosuppressive tumor ecosystem, thus playing an anti-tumor role. They are used widely in clinical treatment; however, the response rate is less than 30% in melanoma 11. At the same time, based on the heterogeneity, complexity, and diversity of tumors, the current treatment strategy for cancer is increasingly inclined toward combination therapy 12. The significant immunostimulating effect of RT has resulted in clinical studies showing that patients who received RT as well as ICI treatment achieved more significant survival benefit than those that received RT alone 13-17. Most studies aimed to activate systemic immune-mediated antitumor effects through radiation-induced *in situ* tumor vaccines 18-20. However, the synergistic effect of combination therapy is not obvious in practice. Therefore, how to improve the efficacy of combination therapy has become an urgent issue in clinical practice.

Many nanomaterials have been developed to overcome radio-resistance and reverse immunosuppression by alleviating hypoxia, activating stimulator of interferon response cGAMP interactor (STING), promoting immune checkpoint blocking, regulating metabolic processes, and remodeling the extracellular matrix (ECM) and fibroblasts 21-24. Activation of the immune system by radiation is largely dependent on the activation of the immune system "accelerator", namely the cyclic GMP-AMP synthase (cGAS)‑STING pathway 25. Radiation therapy exerts its effects by damaging tumor cell DNA structures, leading to abnormal accumulation of DNA damage fragments in the cytoplasm 5. The DNA sensor cGAS recognizes and binds cytoplasmic DNA fragments, catalyzing cGAMP synthesis which then activates STING as a second messenger, triggering downstream signaling cascades that induce potent immune responses 26, 27. However, the activation of the cGAS-STING pathway mediated by these DNA fragments is inherently limited due to the low cytoplasmic transport efficiency of negatively charged DNA fragments, which restricts their binding to cGAS protein 28. Notably, while radiation damages DNA, it also upregulates the DNA exonuclease Trex1, which degrades radiation-induced cytoplasmic DNA, thereby attenuating its immunogenicity 7. Therefore, improving the sensitivity of cGAS recognition of cytosolic dsDNA in DCs using agonists could synergistically benefit cGAS-STING activation to enhance RT and immune checkpoint blockade (ICB) efficacy 30, 31. The metal ion Mn^2+^ is an effective activator of the cGAS-STING pathway, stimulating the production of type I interferon (IFN), significantly promoting the maturation and antigen presentation of DCs and macrophages, enhancing the activation of CD8^+^ T cells, and triggering specific anti-tumor immunity 32, 33. Mn-deficient mice were reported to have severely reduced numbers of tumor-infiltrating cytotoxic CD8^+^ T cells and thus lost control of tumor progression and metastasis 34. More importantly, a completed phase 1 clinical trial (Clinical Trials. Gov. Identifier: NCT03991559) combining Mn^2+^ and anti-programmed cell death 1 (PD-1) antibodies (αPD-1) showed encouraging clinical outcomes in patients with advanced metastatic solid tumors 34. To ensure efficient STING pathway activation, various TME-responsive Mn‑based nanomaterials have been developed as STING nanoagonists 35-38. In addition, Mn is an essential nutritional trace element with well-studied toxicology to human health that plays critical roles in many physiological processes, including innate and adaptive antitumor immune responses 39, 40. Based on these findings, the sustained release of Mn^2+^ as a cGAS‑STING agonist to synchronize with sensitized RT-mediated ICD accumulation offers a potential solution to tackle the above-mentioned challenge in treating solid tumors. ZIF-8 is an X-ray-responsive metal-organic framework (MOF), which is composed of zinc ions coordinated with 2-methylimidazole, with good biocompatibility. ZIF-8 has extremely low systemic toxicity and sensitive pH‑responsive biodegradability; therefore, it can be used as a drug carrier, with wide applications in bioimaging and cancer therapy 41, 42. In addition, a study showed that ZIF-8 has radiological response characteristics, which can achieve controlled drug release 43. Therefore, the development of ZIF-8 frame nanocomposites based on Mn^2+^ is expected to enhance the effect of RT combined with immunotherapy.

Microneedles, which can directly pierce the stratum corneum and deliver drugs to the deep skin layer in a painless manner through microporous channels, have attracted wide attention because of their simple administration, economy, good biocompatibility, and no needle waste 44-47. In the treatment of superficial tumors, MNs can be adapted in size and shape to conform to the irregularities of the tumor lesions, enabling precise drug administration and reduced dosage, which consequently lowers the potential risk of systemic toxicity 48, 49. Additionally, MNs serve as a "mechanical adjuvant", stimulating the release of pre-immunological cytokines in skin tissue 50, 51, thereby enhancing the local immune response and having the potential to augment immunogenic cell death induced by RT.

**Scheme 1:**
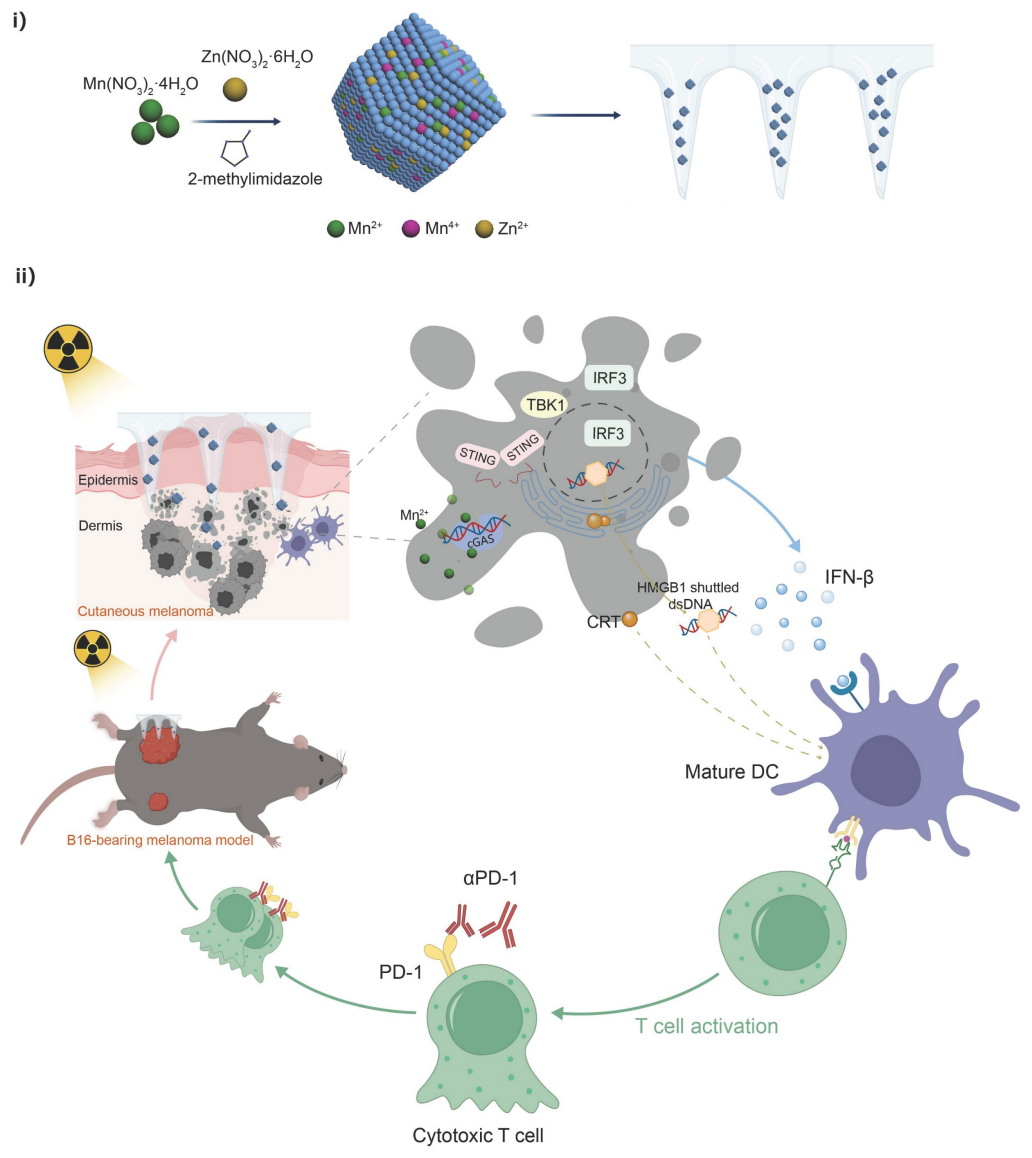
Schematic Illustrations. (i) Schematic illustration of the preparation process of Mn-ZIF-8 and (ii) the proposed mechanism of Mn-ZIF-8-mediated radiosensitization and STING pathway-dependent antitumor immunity for enhanced the radioimmunotherapy efficacy.

Herein, we report that Mn-ZIF-8-loaded MNs act as potent radiosensitizers and cGAS-STING agonists to exert enhanced radio-immunotherapy for cutaneous melanoma (**Scheme 1**). After the MNs are applied to cutaneous melanoma, they rapidly dissolve and release Mn-ZIF-8 nanoparticles (NPs), which act as radiosensitizers to induce ICD and accumulate DNA damage in the tumor. After radiation, Mn^2+^ is continuously released into tumor tissues to promote DC maturation by activating the cGAS-STING cascade signaling pathway, including inducing the phosphorylation of STING and interferon regulatory factor 3 (IRF3), and upregulating interferon beta (IFN-β) expression. The maturation of DCs and integration with ICB further evokes robust antitumor immunity to combat primary and metastatic tumors. Therefore, efficient radiosensitization synchronized with a cGAS-STING pathway stimulation‑based immunoregulation strategy is highly likely to optimize cancer radioimmunotherapy in clinical practice.

## Results

### Synthesis and Characterization of Mn-ZIF-8

**Figure 1:**
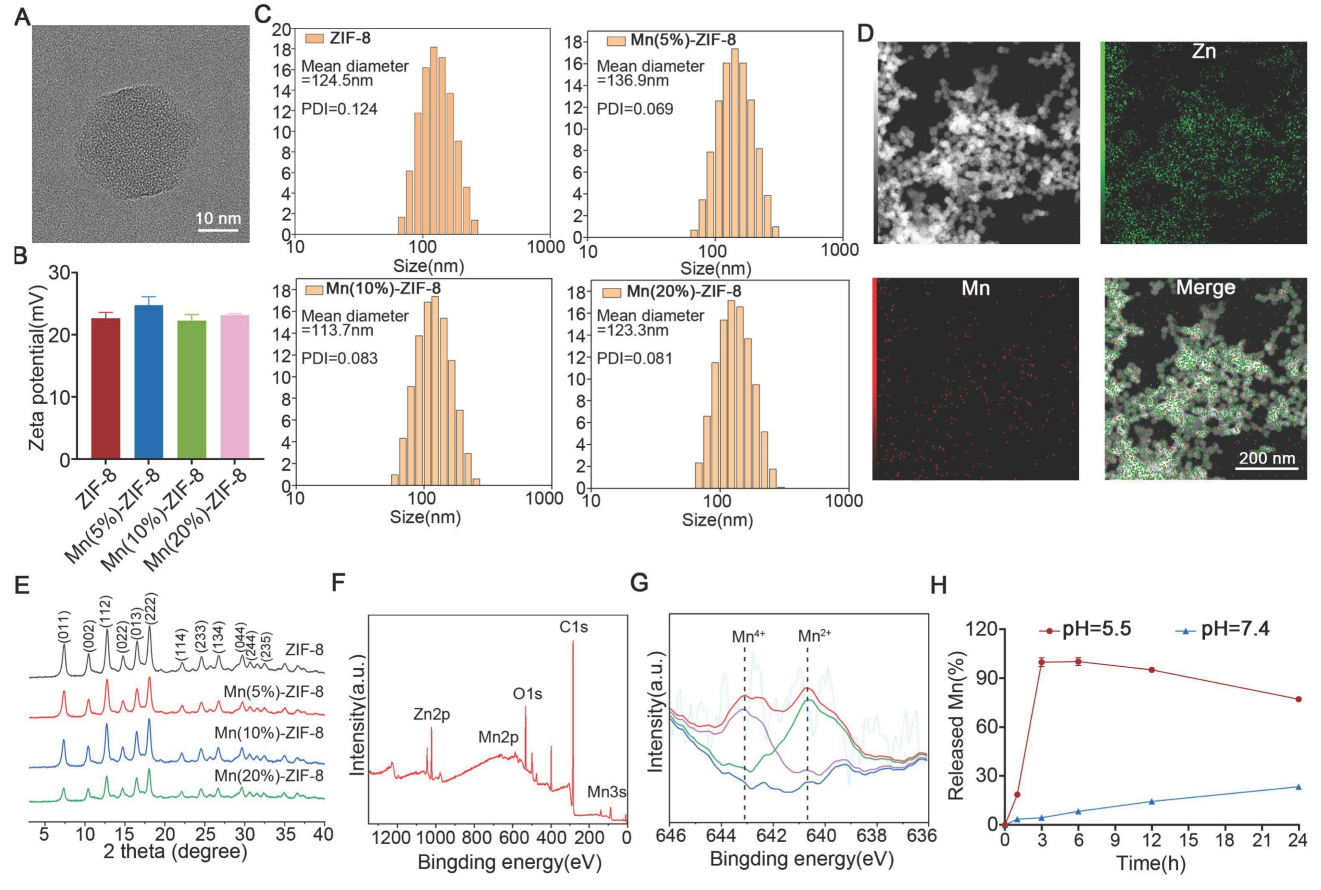
Construction and characterization of Mn-ZIF-8. (A) TEM images of Mn(20%)-ZIF-8 at different magnifications. (B) Zeta potentials and (C, *n* = 3 per group) Hydrodynamic diameters of ZIF-8 and Mn-ZIF-8. (D) Elemental mapping images of Mn(20%)-ZIF-8. (E) The XRD patterns of ZIF-8 and Mn-ZIF-8. (F) The XPS survey spectra of Mn(20%)-ZIF-8. (G) The XPS spectra of Mn 2p. The blue line represents the fitted baseline, the purple and green lines correspond to the fitted peaks for Mn^4+^ and Mn^2+^, and the red line shows the final fitted curve. (H) The cumulative release profile of Mn^2+^ from Mn-ZIF-8 MNs under different pH conditions. The data are presented as the mean ± SD.

The preparation process of Mn-ZIF-8 is shown in Scheme 1 and Figure S1. The ZIF-8 skeleton was modified by doping Mn^2+^ and Mn^4+^ ions, resulting in the synthesis of Mn‑ZIF-8. The transmission electron microscopy (TEM) images revealed that ZIF-8 retained its original structure of a rhomboid dodecahedron shape after modification with Mn^2+^ and Mn^4+^ (Figure 1A). The zeta potentials (Figure 1B) were almost consistent and indicating that Mn ion doping did not change the basic properties of the ZIF-8 NPs and the hydrodynamic diameters of ZIF-8 and Mn-ZIF-8 (Figure 1C) were 124.5, 136.9, 113.7, and 123.3 nm. The Mn and Zn elements were distributed throughout the whole NPs (Figure 1D). The area of Mn equated to a doping rate of 20%, which was consistent with the theoretical value, and the elemental ratio of Mn:Zn was determined to be 96.38:3.62. Consistent with a previous report 52, the X-ray diffraction (XRD) patterns of ZIF-8 and Mn-ZIF-8 revealed characteristic diffraction peaks (Figure 1E). Moreover, with an increased Mn doping rate, the main peak (011) of Mn-ZIF-8 gradually shifted to the right, probably because of the replacement of Zn ions with Mn in the ZIF-8 skeleton. X-ray photoelectron spectroscopy (XPS) was conducted to analyze the chemical composition and valence states of Mn. Figure 1F shows the main corresponding peaks of Zn, Mn, and O, and the Mn 2p XPS spectra are shown in Figure 1G. The peak of Mn 2p_3/2_ could be divided into two characteristic peaks (640.7 and 642.8 eV), which were consistent with the reported data for Mn^2+^ and Mn^4+^, respectively. This suggests that Mn^2+^ and Mn^4+^ can be found in the structure of Mn‑ZIF‑8: the ratio of Mn^2+^ was 50.05% and the ratio of Mn^4+^ is 49.95%. As Figure 1H shown, at pH = 5.5, Mn^2+^ was nearly completely released within approximately 3 h, demonstrating that Mn-ZIF-8 MNs can rapidly release Mn^2+^ in the acidic tumor microenvironment to exert antitumor effects. In contrast, at pH = 7.4, the cumulative release rate after 24 h was only about 23%, suggesting minimal leakage into normal tissues to cause undesired side effects.

### Anticancer Effect and Radiosensitization of Mn-ZIF-8 *In Vitro*

**Figure 2:**
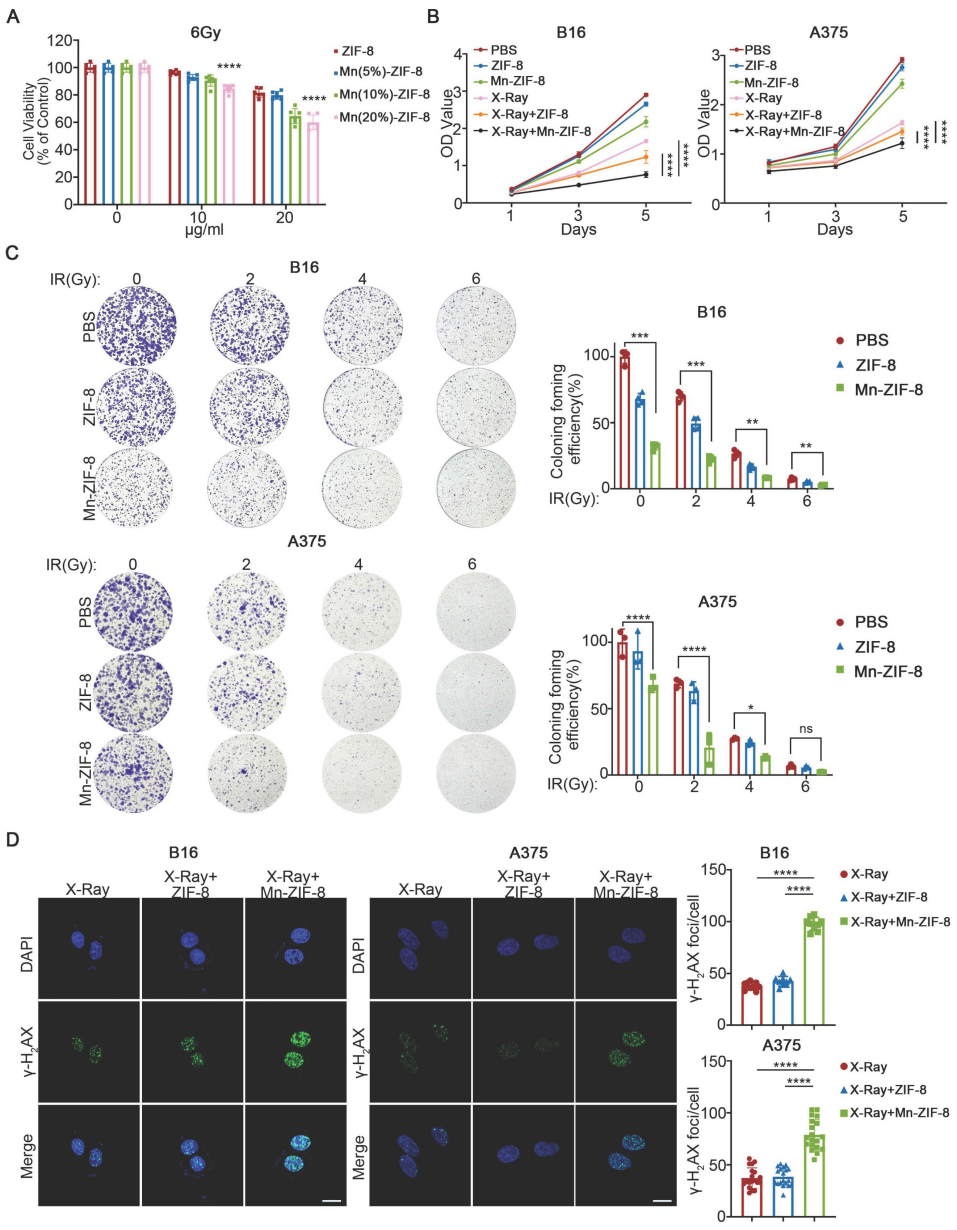
Effect of Mn-ZIF-8 on melanoma cell proliferation and dsDNA damage. (A) Effect of Mn-ZIF-8 at different concentrations and its components in combination with radiotherapy (6 Gy) on the viability of B16 cells (*n* = 6 per group). (B) Effect of PBS, ZIF-8, and Mn‑ZIF-8 in combination, with or without radiotherapy (B16, 6 Gy; A375, 4Gy), on the proliferation of melanoma cells, as assessed using the CCK8 assay (*n* = 5 per group). (C) Effect of PBS, ZIF-8, and Mn-ZIF-8 in combination, with or without different doses of radiotherapy (2, 4, and 6 Gy), on the proliferation of melanoma cells, as assessed using a colony formation assay (B16, *n* = 4 per group; A375, *n* = 3 per group). (D) Effect of PBS, ZIF-8, and Mn-ZIF-8 in combination, with radiotherapy (2Gy), on the dsDNA breaks in melanoma cells, as evaluated using immunofluorescence staining of γ-H_2_AX. Scale bar = 10 μm. The data are presented as the mean ± SD. *P < 0.05, **P < 0.01, ***P < 0.001, ****P < 0.0001.

Initially, we evaluated the impact of Mn-ZIF-8 and its constituent elements at varying concentrations, in conjunction with a 6Gy X-ray irradiation, on the survival rate of B16 cells (epithelial-like cells isolated from skin of a mouse with melanoma). We used the CCK-8 assay to evaluate the radiosensitization efficiency of Mn-ZIF-8 with different Mn^2+^ doping ratios (5%, 10%, and 20%). The experimental data clearly show that at all tested radiation doses (0, 2, 4, and 6 Gy), Mn(20%)-ZIF-8 at 20 μg/mL exhibited the best radiosensitization effect, with significantly lower cell survival rates compared to the 5% and 10% doping groups (Figure 2A and Figure S2). This dose-dependent enhancement confirms a positive correlation between Mn^2+^ content and radiosensitization efficacy. Furthermore, under 6 Gy irradiation, we treated B16 cells with different doping ratios of Mn-ZIF-8 (20 μg/mL) and measured key proteins involved in the STING pathway. The Western blot results demonstrated that the 20% Mn-ZIF-8 group induced higher levels of p-STING and p-IRF3 expression compared to the 5% and 10% groups, suggesting that a higher Mn content more effectively activates the cGAS-STING pathway (Figure S3). Therefore, ZIF-8 and Mn-ZIF-8 at 20 μg/mL (referred to Mn(20%)-ZIF-8) were chosen for further investigation. Mn-ZIF-8 exhibited significant cytotoxicity after irradiation (IR) treatment in B16 and A375 melanoma cells, as shown by cell couniting kit-8 (CCK8) assays (Figure 2B). Furthermore, we conducted clonogenic assays using human keratinocyte HaCaT cells to assess the biocompatibility of Mn-ZIF-8. Treatment with 20 μg/mL Mn-ZIF-8 demonstrated no significant reduction in colony formation efficiency compared to PBS control (Figure S4). This result clearly indicates that Mn-ZIF-8 exhibits minimal toxicity toward normal cells. By contrast, combined Mn-ZIF-8 and IR treatment could effectively inhibit colony formation of B16 and A375 melanoma cells (Figure 2C). Then, the DNA damage induced by IR in B16 and A375 melanoma cells was evaluated using immunofluorescence staining of γ-H_2_AX. Mn-ZIF-8 markedly increased the formation of IR-induced γ-H_2_AX foci (Figure 2D). Taken together, these results indicated Mn-ZIF-8 could increase the sensitivity of melanoma cells to RT and induce obvious DNA damage under RT.

### Mn-ZIF-8 Enhanced ICD and the Activation of the STING Pathway Induced by RT *In Vitro*

**Figure 3:**
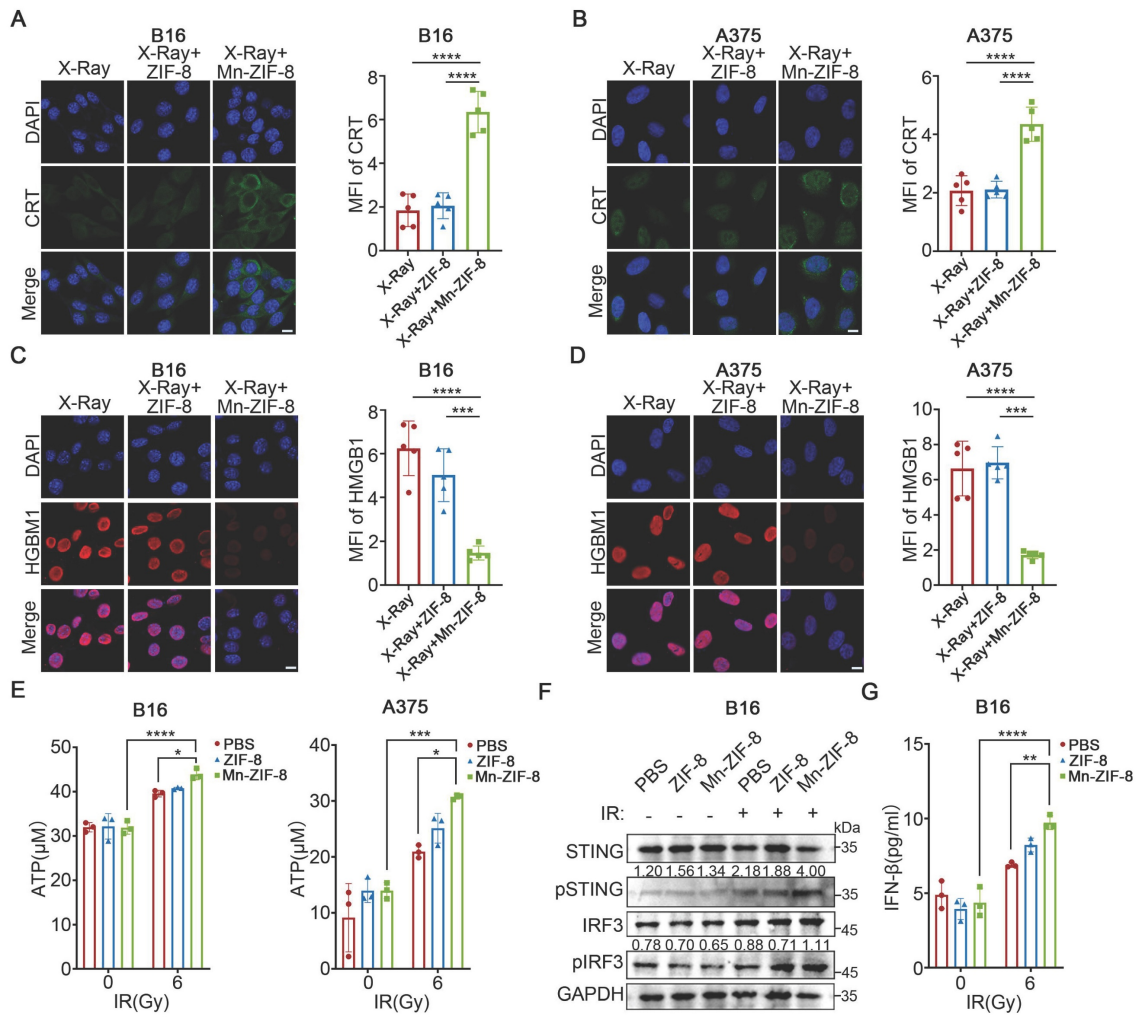
Mn-ZIF-8 enhanced ICD and the activation of the STING pathway induced by RT *in vitro*. (A) and (B) Quantification of CRT fluorescence intensities and representative fluorescence images of melanoma cells subjected to various treatments (*n* = 5 per group). (C) and (D) Quantification of HMGB1 fluorescence intensities and representative fluorescence images of melanoma cells subjected to various treatments (*n* = 5 per group). Scale bar = 10 μm. (F) Western blotting analysis of the activation of cGAS-STING in melanoma cells subjected to various treatments. ELISA analysis of the secretion of (E) ATP and (G) IFN-β from melanoma cells subjected to with various treatments (*n* = 3 per group). The data are presented as the mean ± SD. *P < 0.05, **P < 0.01, ***P < 0.001, ****P < 0.0001.

Radiotherapy can activate the immune system against tumors by inducing ICD, a specific cell death modality, which would trigger the release of damage-associated molecular patterns (DAMPs), such as calreticulin (CRT) exposure, high mobility group box 1 (HMGB1) release, and ATP secretion, thereby increasing the immunogenicity of the TME 53, 54. Therefore, we investigated the effects of Mn-ZIF-8 on RT-induced ICD by examining CRT exposure, HMGB1 release, and ATP secretion. Significant expression of CRT was observed after treatment with Mn-ZIF-8 in combination with IR (6 Gy), in sharp contrast to other groups (Figure 3A-B). Furthermore, treatment with Mn-ZIF-8 in combination with IR (6 Gy) enhanced the release of HMGB1 from the cell nuclei as well as ATP production in B16 and A375 melanoma cells (Figure 3C-E). These results indicated that treatment with Mn-ZIF-8 in combination with RT would significantly promote the ICD of tumor cells, which is a prerequisite for a subsequent antitumor immune response. Additionally, we also observed that Mn-ZIF-8 treatment combined with X-ray irradiation (6 Gy) significantly enhanced mitochondrial superoxide levels in B16 cells compared to either X-ray alone or X-ray + ZIF-8 treatments, as quantified by mitochondrial superoxide fluorescence intensity measurements (Figure S5). This confirms that Mn-ZIF-8 can potentiate radiotherapy through enhanced ROS generation.

The STING pathway is intricately linked to the generation of the antitumor immune response. It has been established that free Mn^2+^ ions significantly amplify cGAS‑STING signaling cascade activation, exerting a comprehensive effect, ranging from boosting the synthesis of cGAMP to enhancing cGAMP-STING binding affinity 55, 56. Next, we evaluated the capacity of Mn-ZIF-8 to activate the STING pathway *in vitro*. STING pathway-related proteins were examined using western blotting. As shown in Figure 3F, increased levels of phosphorylated STING and IRF3 were observed after treatment with Mn-ZIF-8 in combination with IR (6Gy), indicating that Mn‑ZIF‑8 contributed to activation of the STING pathway in melanoma cells induced by RT. Moreover, Mn-ZIF-8 significantly promoted IFN-β secretion after IR treatment, further indicating effective activation of STING pathway by Mn-ZIF-8 (Figure 3G).

### Fabrication and Characterization of Mn-ZIF-8 MNs

**Figure 4:**
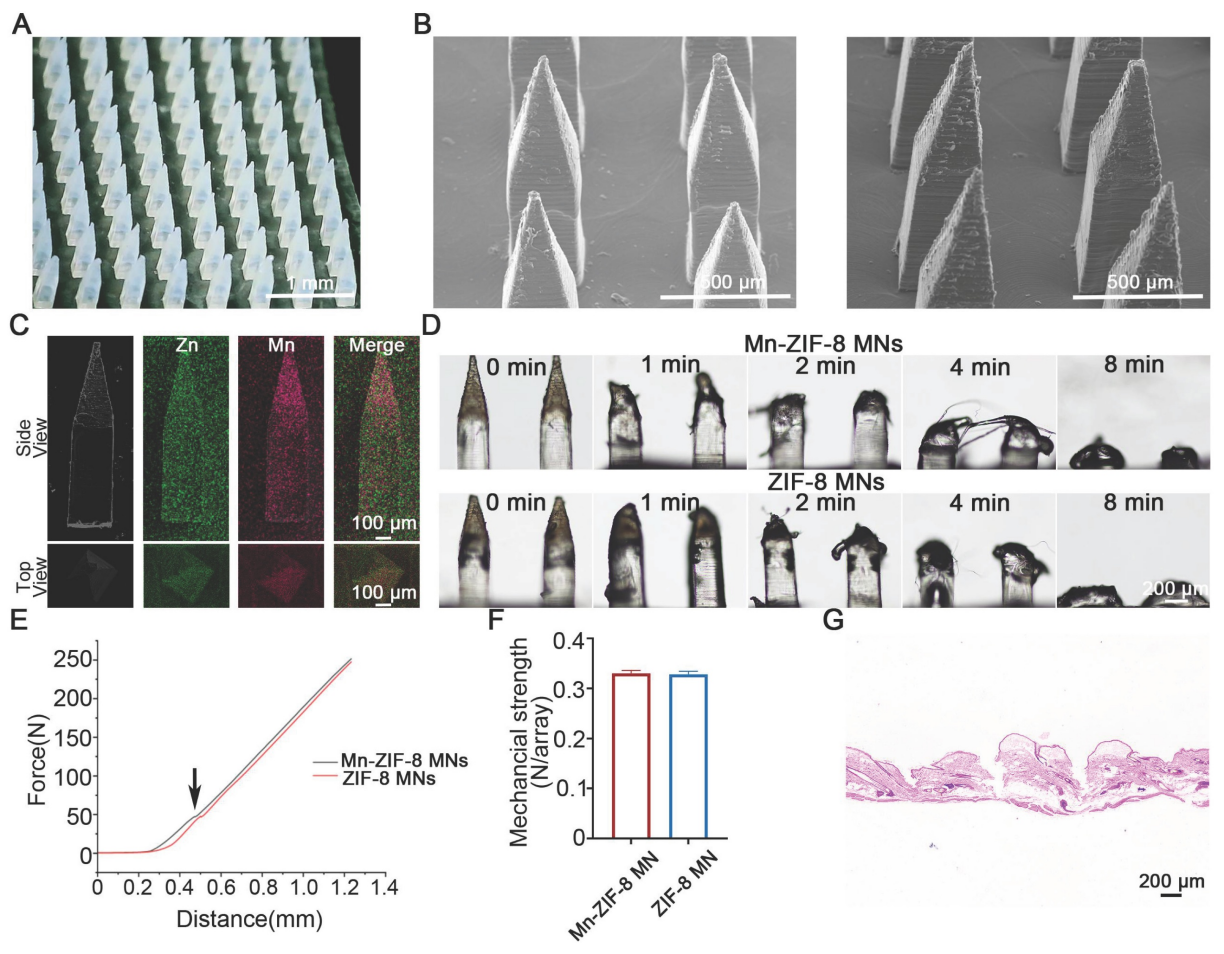
Construction and characterization of MNs loaded with Mn-ZIF-8. (A) Photograph of the Mn(20%)-ZIF-8 MNs array. (B) SEM images of Mn(20%)-ZIF-8 and ZIF-8 MNs. (C) Main view and top view of elemental mapping images of Mn(20%)-ZIF-8 MNs. (D) Solubility of Mn(20%)-ZIF-8 MNs. (E) The force-displacement curves of Mn(20%)-ZIF-8 and ZIF-8 MNs. Arrow: Fracture point of MNs. (F) Mechanical strength of the force per individual needle (N per needle) (*n* = 3 per group). (G) H&E staining of the rat skin punctured with Mn(20%)-ZIF-8 MNs. The data are presented as the mean ± SD.

Centrifugal micro-perfusion method was used to prepare the MNs (Figure S6). The resultant Mn-ZIF-8 MNs and ZIF-8 MNs were pyramid‑shaped and regularly arranged in a 12 × 12 array (Figure 4A and Figure S7). The MNs had a needle height of 1200 μm, a base width of 300 μm, and a tip-to-tip interspace of 800 μm, located on a 1 × 1 cm patch (Figure 4B and Figure S8). In addition, elemental mapping of the main and top (Figure 4C) views of the MNs was scanned to study the distribution of Mn and Zn ions in the MNs. The majority of Mn and Zn ions were observed to be distributed in the needle tips, probably owing to the concentration of Mn-ZIF-8 in the needle tips under centrifugal force. Next, we further validated the stability of the microneedles during both preparation and storage processes. First, we conducted morphological comparisons between freshly prepared Mn-ZIF-8 MNs and Mn-ZIF-8 MNs after storage (Figure S9). The results showed that the structural morphology of Mn-ZIF-8 nanoparticles was well preserved, without signs of aggregation, deformation, or disintegration, indicating excellent physical stability during fabrication and storage. Second, TEM-EDS elemental analysis demonstrated that the elemental ratios of Zn and Mn remained consistent between the fresh and stored Mn-ZIF-8 MNs, further confirming that the chemical composition and doping structure of Mn-ZIF-8 were not altered throughout the microneedle preparation process (Figure S10). The morphological changes of Mn-ZIF-8 MNs were recorded using optical microscopy, which showed that the MNs were completely dissolved after 8 min of application (Figure 4D). The dissolution behavior of MNs was conducive to the release and diffusion of the drug from the MNs, thereby increasing drug delivery efficiency. The mechanical properties of MNs are a key factor that determines their skin insertion ability. The average fracture forces of Mn-ZIF-8 MNs and ZIF-8 MNs were 0.3305 N/needle and 0.3285 N/needle, respectively, indicating that the fracture forces of both MNs are greater than the minimum force (0.1N/needle) required for the MNs to pierce the stratum corneum (Figure 4E-F) 57. The mechanical strength of blank MNs was 0.3052 N per needle which was lower than that of Mn-ZIF-8 MNs and ZIF-8 MNs (Figure S11). The enhanced mechanical strength of Mn-ZIF-8 MNs and ZIF-8 MNs may be attributed to the electrostatic interactions between the positively charged Mn-ZIF-8/ZIF-8 and negatively charged hyaluronic acid. Simultaneously, we captured morphological images of the microneedles before and after mechanical testing (Figure S12). Hematoxylin and eosin (H&E) staining was performed on rat skin receiving microneedle puncture. Obvious micropores with a depth of 300-340 microns were observed in the H&E-stained skin tissue, indicating that the prepared MNs possess favorable skin penetration capabilities and can successfully deliver drugs to the dermis (Figure 4G and Figure S13). We captured fluorescence images (Figure S14) of IR780-labeled Mn-ZIF-8 microacupuncture at different times after subcutaneous tumor insertion. The drug delivery efficiency is a key factor affecting therapeutic outcomes. Therefore, we compared the drug delivery efficiency of microneedles and intratumoral injection by monitoring the biodistribution of fluorescence-labelled Mn-ZIF-8 nanoparticles. The fluorescence intensity (representing drug retention and sustained release) after intratumoral injection increased rapidly, peaking within 6 hours post-administration, followed by a sharp decline, indicating rapid drug clearance from the tumor site. In stark contrast, MN administration showed a markedly different pharmacokinetic profile: the fluorescence intensity remained relatively stable and high for at least 24 hours post-administration (over 50% fluorescence retention), and subsequently declined slowly, maintaining measurable intensity even at 120 hours (Figure S15).

### Antitumor Effects of Mn-ZIF-8 MNs Combined with RT in a B16 Melanoma Xenograft Mouse Model

**Figure 5:**
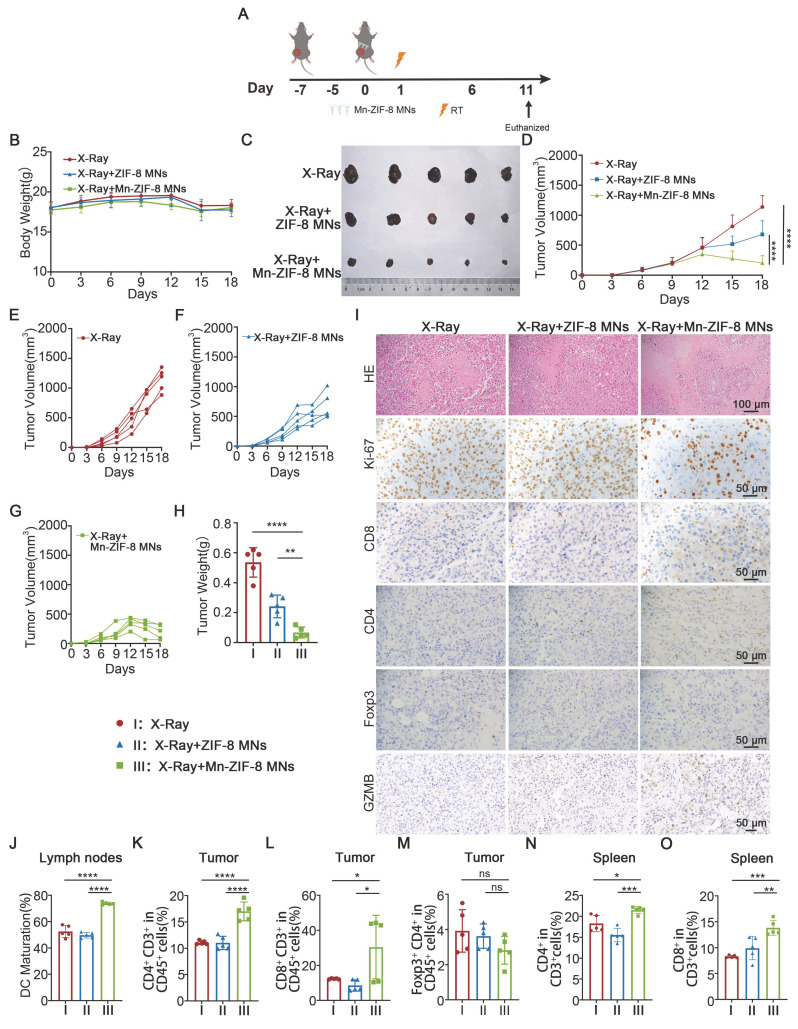
Antitumor effects of Mn-ZIF-8 MNs in a B16 melanoma xenograft mouse model. (A) Experimental timeline for the treatment of B16 tumor-bearing C57BL/6J mice. (B) Body weight curve of mice during treatment. (C) Photograph of B16 tumors isolated from the mice on day 18. (D) Tumor growth curve of mice subjected to different treatments. (E) and (F) and (G) Individual tumor growth curves of mice after different treatments. (H) Weights of tumors isolated from the mice on day 18. (I) H&E staining of tumor tissues and IHC images showing Ki67, CD4^+^ T, and CD8^+^ T cell infiltration, Foxp3 and GZMB expression after the indicated treatments. (J) Quantitative analysis of mature DCs (CD80^+^ CD86^+^ in CD11c^+^ cells) in inguinal lymph nodes adjacent to tumors after treatment. (K-M) Percentages of tumor-infiltrating CD4^+^ T, CD8^+^ T, and Treg cells. (N) and (O) Percentages of spleen-infiltrating CD4^+^ T and CD8^+^ T cells. The data are presented as the mean ± SD; *n* = 5 per group. *P < 0.05, **P < 0.01, ***P < 0.001, ****P < 0.0001.

To evaluate the anticancer efficacy of Mn-ZIF-8 prodrugs delivered by MNs patches, we implanted B16 melanoma cells subcutaneously in the right lateral thigh area of C57BL/6J mice. Once the tumor reached approximately 100 mm^3^, the mice were randomly divided into three groups (n = 5): X-ray (I), X-ray + ZIF-8 MNs (II), and X‑ray + Mn-ZIF-8 MNs (III). On the seventh day after tumor implantation, the MNs patches were applied to the tumor site and the tumors were irradiated with X-rays (12Gy) once at 16 hours after Mn-ZIF-8 MNs administration (Figure 5A). The tumor volumes and body weights were monitored every 3 days from tumor implantation until the mice were euthanized. All mice showed a slight weight loss about a week after RT and remained within the normal body weight range during the treatments (Figure 5B). Major organs (heart, liver, spleen, lung, and kidney) from mice were analyzed by H&E staining (Figure S16). No obvious tissue damage or side effects were found in the mouse organs, indicating excellent biosafety. Furthermore, a complete blood panel analysis and serum biochemistry assay (Figure S17) were performed. Notably, almost all the examined indexes were in the normal ranges, suggesting no obvious systematic toxic side effects of the treatment.

Tumor growth was only slightly delayed in the X-ray + ZIF-8 MNs group. However, the tumor growth in the X-ray + Mn-ZIF-8 MNs group was significantly delayed (Figure 5C-H). Mice receiving different treatments were sacrificed on the 16th day after tumor implantation and the collected tumors were sliced for immunohistochemistry (IHC) staining or dissociated into cell suspensions for flow cytometry analysis. Immunohistochemical staining for marker of proliferation Ki-67 (Ki-67) and H&E staining in tumor slices showed that the most cell death and the least cell proliferation occurred in the X-ray + Mn-ZIF-8 MNs treatment group (Figure 5I), further revealing the Mn-ZIF-8 MNs-induced RT enhancement.

Relevant studies have shown that RT-induced ICD of tumor cells and STING pathway activation can activate DCs and further enhance anti-tumor immune activity. We demonstrated *in vitro* that Mn-ZIF-8 can promote IR-induced ICD and STING pathway activation. Then, we verified the underlying mechanism of the antitumor responses triggered by treatment with X-ray + Mn-ZIF-8 MNs combined with RT *in vivo*. First, the IHC results indicated that X-ray + Mn-ZIF-8 MNs treatment drove much higher CD4^+^ T cell infiltration and exhibited the highest level of CD8^+^ T cells, but no significant difference of the infiltration of regulatory T cells (Tregs) was found in tumors. We examined the expression of granzyme B (GZMB)—a key effector molecule of activated cytotoxic T cells for tumor killing 58. The Mn-ZIF-8 MNs + X-ray group showed significantly higher GZMB-positive areas compared to X-ray alone and ZIF-8 MNs + X-ray, which correlates with our observed increase in CD8^+^ T cell infiltration (Figure 5I and Figure S18). The maturation of DCs in the inguinal lymph nodes was detected using flow cytometry. The mice treated with X-ray + Mn-ZIF-8 MNs could effectively enhance DC maturation in lymph nodes, thus enhancing their antigen presentation ability (Figure 5J and Figure S19). Later, the proportions of CD4^+^ T cells (Figure 5K and Figure S20), CD8^+^ T cells (Figure 5L and Figure S21) and Tregs cells (Figure 5M and Figure S22) in tumors were also measured using flow cytometry, and the results were approximately coincident with the IHC results. Meanwhile, treatment with X-ray + Mn-ZIF-8 MNs increased the infiltration of CD8^+^ T cells and CD4^+^ T cells in the spleen (Figure 5N-O and Figure S23-24). Collectively, X-ray + Mn-ZIF-8 MNs could promote DC maturation and CD8^+^ T cell infiltration, thus inducing robust systemic antitumor immunity *in vivo*.

### Mn-ZIF-8 MNs Plus RT Potentiates Systemic Antitumor Immunity Induced by ICB

**Figure 6:**
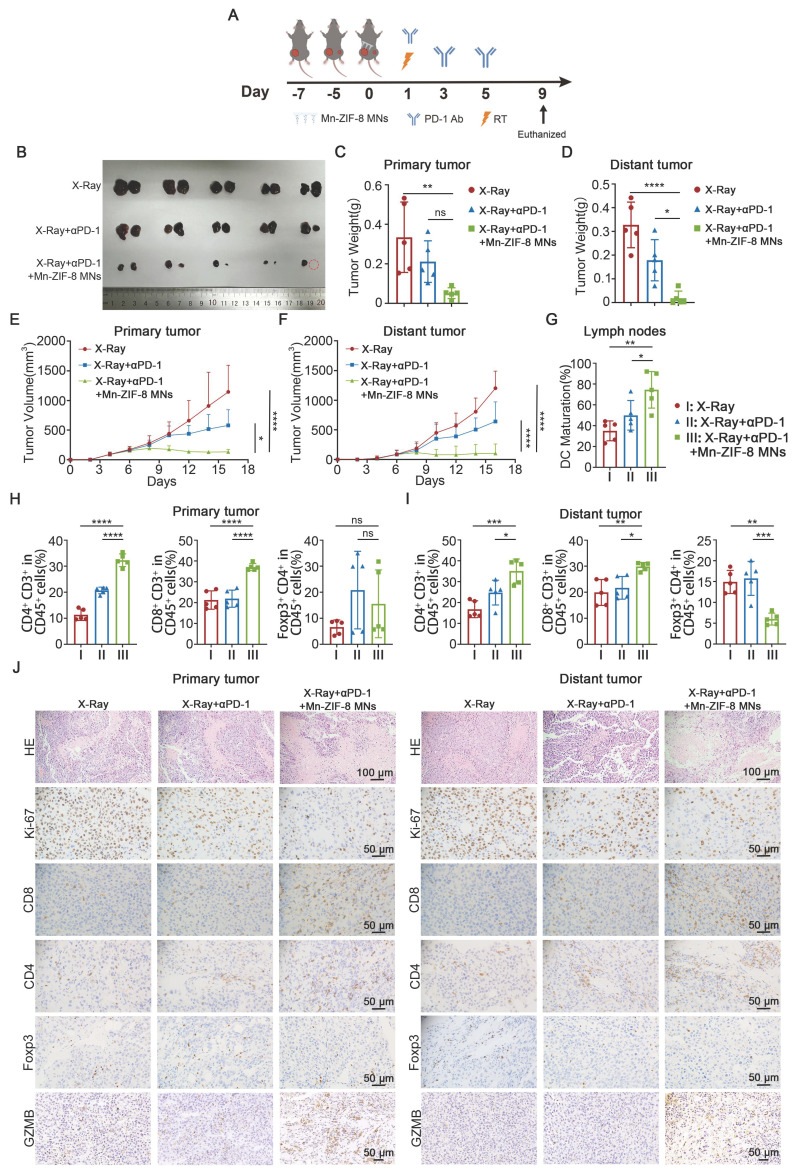
Mn-ZIF-8 MNs combined with RT plus ICB elicited systemic antitumor immunity. (A) Experimental timeline for the treatment of bilateral B16 tumor-bearing C57BL/6J mice. (B) Photograph of primary (left) and metastatic (right) B16 tumors isolated from the mice on day 16. (C) and (D) Weights of primary tumors and distant tumors isolated from the mice on day 16. (E) and (F) Primary tumors and distant tumor growth curves of the mice after different treatments. (G) Quantitative analysis of mature DCs (CD80^+^ CD86^+^ in CD11c^+^ cells) in inguinal lymph nodes adjacent to primary tumors after treatment. (H) Percentages of primary tumor-infiltrating CD4^+^ T, CD8^+^ T, and Treg cells. (I) Percentages of distant tumor-infiltrating CD4^+^ T, CD8^+^ T, and Treg cells. (J) H&E staining of bilateral tumor tissues and IHC images showing Ki67, CD4^+^ T, and CD8^+^ T cell infiltration, and Foxp3 and GZMB expression after the indicated treatments. The data are presented as the mean ± SD; *n* = 5 per group. *P < 0.05, **P < 0.01, ***P < 0.001, ****P < 0.0001.

To investigate the systemic immune responses and the therapeutic potential of X‑ray + Mn-ZIF-8 MNs combined with ICB, we established a bilateral B16 subcutaneous tumor model on C57BL/6J mice. B16 cells were subcutaneously injected into the left side of mice 2 days after the inoculation of primary tumors in the right side. Once the right tumor reached approximately 100 mm^3^, the mice were randomly divided into three groups (n = 5): X‑ray (I), X-ray + αPD-1 (II), and X-ray + αPD-1 + Mn-ZIF-8 MNs (III). On the seventh day after tumor implantation, the MNs patches were applied to the primary tumor site and the primary tumors were irradiated with X-rays (12Gy) once at 16 hours after application. The mice of groups II and III were intraperitoneally (i.p.) injected with αPD-1 (10 mg/kg) on days 8, 10, and 12 after tumor implantation (Figure 6A). The tumor volumes and body weights were monitored every 2 days from tumor implantation until the mice were euthanized. The primary and distant tumor growth were recorded and analyzed. Treatment with X-ray + αPD-1 + Mn-ZIF-8 MNs showed the strongest growth control of both primary and distant tumors compared with that of the other groups (Figure 6B-F and Figure S25).

Next, mice receiving different treatments were sacrificed on the 16th day after tumor implantation and the collected primary and distant tumors were sectioned for IHC staining or dissociated into cell suspensions for flow cytometry analysis. First, the maturation of DCs in the inguinal lymph nodes on the primary tumor side was detected using flow cytometry. The mice treated with X-ray + αPD-1 + Mn-ZIF-8 MNs showed effective enhancement of DC maturation in their lymph nodes, thus enhancing the antigen presentation ability (Figure 6G and Figure S26). The flow cytometry results indicated that treatment with X-ray + αPD-1 + Mn-ZIF-8 MNs drove much higher infiltration of CD4^+^ T and CD8^+^ T cells in the primary tumors; however, no significant difference of the infiltration of Tregs in the primary tumors was found for three groups (Figure 6H and Figure S27-29). The findings regarding CD4^+^ T and CD8^+^ T cell populations in distant tumors closely mirrored those observed in the primary tumors (Figure 6I and Figure S30-31). However, treatment with X-ray + αPD-1 + Mn-ZIF-8 MNs reduced the percentage of Tregs in the distant tumors (Figure 6I and Figure S32). Later, the proportions of CD4^+^ T cells, CD8^+^ T cells, and Tregs cells in the primary and distant tumors were also measured using IHC, and the results were approximately coincident with the flow cytometry results (Figure 6J and Figure S33). Likewise, the X-ray + αPD-1 + Mn-ZIF-8 MNs group demonstrated significantly enhanced GZMB expression in both primary and distant tumors compared to other groups (Figure 6J and Figure S33). Consistently, H&E and immunohistochemical Ki67 staining of primary and distant tumor slices showed the most cell death and the least cell proliferation in the group treated with X-ray + αPD-1 + Mn-ZIF-8 MNs (Figure 6J and and Figure S33). Collectively, these results illustrated that Mn-ZIF-8 MNs combined with X-ray treatment triggered a strong systemic immune response, which effectively synergized with ICB to eliminate both primary and metastatic tumors.

## Discussion and Conclusion

In summary, we proposed a rapidly dissolving MNs patch loaded with high bioactivity molecular sieve imidazole skeleton sealed with Mn^2+^ nanoparticles. we specifically demonstrated that Mn-ZIF-8-based microneedles potentiate radiation-induced ICD and enhance activation of the cGAS-STING pathway, leading to increased infiltration of cytotoxic CD8⁺ T cells and maturation of dendritic cells—key features of an effective anti-tumor immune response. These immunological mechanisms are not unique to melanoma but are shared across many “cold” tumors that exhibit limited baseline immune infiltration, such as breast cancer, pancreatic cancer, and colorectal cancer 40. Indeed, STING agonists have been investigated as broad-spectrum immune adjuvants in multiple solid tumor models beyond melanoma 59. Additionally, our delivery platform—X-ray-responsive Mn-ZIF-8 nanoparticles in dissolving microneedles—was engineered to provide localized radiosensitization and immune modulation, a strategy applicable to many superficial or accessible solid tumors. For example, squamous cell carcinoma, head and neck cancers, and cutaneous metastases from breast or gynecologic cancers are all relevant clinical targets where localized treatment via MNs could be readily adapted 60. Therefore, the Mn‑ZIF-8-loaded MNs demonstrated significant potential to improve the efficacy of radio-immunotherapy in cutaneous melanoma. The potential clinical applications of Mn-ZIF-8 MNs are particularly compelling. Mn^2+^ ions have already demonstrated promising results in clinical trials for solid tumors as STING pathway agonists, further validating the clinical relevance of Mn-based therapies 34. ZIF-8 itself, due to its excellent biocompatibility, and biodegradability, has also emerged as a clinically relevant carrier platform, enhancing therapeutic precision and controlled drug release 61.

Additionally, microneedle systems have been advancing into clinical trials, particularly for dermatological diseases and vaccines, establishing a solid foundation for clinical adoption due to their ease of use, patient compliance, and reduced biohazard risk compared to traditional injections 62. However, despite these promising aspects, significant translational challenges remain. These include ensuring consistent batch-to-batch quality and reproducibility of biomaterials, optimizing large-scale manufacturing processes, and establishing robust sterilization methods without compromising therapeutic efficacy 63. Moreover, regulatory pathways for combination products involving novel nanomaterials (like ZIF-8 and metal-ion-based therapeutics) and delivery systems (like MNs) require extensive validation and clinical safety profiles, which are currently limited and require thorough evaluation 64. Long-term biocompatibility, biodegradation kinetics, potential immunogenicity, and off-target effects also represent significant considerations before clinical implementation.

## Experimental Section

### Materials

Mn(NO_3_)_2_·4H_2_O, Zn(NO_3_)_2_·6H_2_O, Methanol, Ethanol, Gelatin, and IR780 were obtained from Macklin Industrial, Inc. (Shanghai, China). 2‑methylimidazole was obtained from Aladdin Industrial, Inc. (Shanghai, China). Hyaluronic acid (HA) and PVP K90 were purchased from BASF (Ludwigshafen, Germany).

### Synthesis of Mn-ZIF-8 NPs

We weighed out 0.5 mmol (20%), 0.25 mmol (10%) or 0.125 mmol (5%) of Mn(NO_3_)_2_·4H_2_O, and 2 mmol of Zn(NO_3_)_2_·6H_2_O, and dissolved them together in 20 mL of methanol to obtain mixed solution 1. We weighed out 40 mmol of 2-methylimidazole and dissolved it in 80 mL of methanol. Using a pipette, mixed solution 1 was added slowly to the 2-methylimidazole methanol solution under gentle magnetic stirring at room temperature. After the addition was complete, the mixture was stirred at room temperature for 4 h, and then then placed at 50 °C for 1 h. The mixture was centrifuged at 13000 g at room temperature for 10 minutes, the precipitate was washed with methanol twice, and then concentrated to obtain 20 mL of Mn-ZIF-8 NPs. Compositions of ZIF-8 and Mn-ZIF-8 are provided in Supporting Information: Table S4.

### Synthesis of ZIF-8 NPs

We weighed out 2.5 mmol of Zn(NO_3_)_2_.6H_2_O and dissolved it in 20 mL of methanol to obtain Solution 1. The remaining steps are the same as those in *Synthesis of Mn-ZIF-8 NPs*.

### Fabrication and Characterization of Mn-ZIF-8 MNs

All MNs in this study were prepared using a polydimethylsiloxane (PDMS) micromold. The needle tips were fabricated using a 1:1 (v/v) mixture of 350 mg/mL hyaluronic acid solution and Mn-ZIF-8 methanol solution. The base layer was prepared from polyvinylpyrrolidone (PVP K90) ethanol solution (312.5 mg/mL). 200 μL of the needle solution was dispensed into each PDMS mold well, followed by centrifugation (2,080 × g, 5 min, 4-10 °C) to ensure complete microchannel filling. Residual solution on the mold surface was removed using an aluminum scraper. A second centrifugation (2,080 × g, 30 min, 4-10 °C) was performed to compress the needle matrix and initiate partial drying. 300 μL of base solution was added to each well and centrifuged (2,080 × g, 5 min, 4-10 °C) for uniform distribution. The male mold was air-dried at room temperature for 48 h. The resulting Mn-ZIF-8 MNs were then carefully demolded and stored in a desiccator until use.

### Mechanical characteristics of the MNs

The mechanical properties of the MNs array were evaluated using a Texture analyzer (Stable micro systems, UK). Each MNs patch tested was attached to a flat plate with the tip of the needle facing up. A force perpendicular to the plate was applied at a constant speed of 0.5 mm/min and the compression distance was set to 1 mm.

### Insertion capacity of MNs

MNs were placed vertically on the skin surface of the abdomen of SD rats, the MNs base layer was pressed vertically for 2 min. The MNs were then removed, the skin at the administration site was clipped, and immediately soaked in 4% paraformaldehyde fixing solution. H&E staining was performed after paraffin-embedded skin sections were obtained.

### *In Vitro* Degradation Experiment

Mn-ZIF-8 MNs was placed into a dialysis bag (MWCO=3500) and injected with 2 mL of PBS (pH=5.5/pH=7.4). After sealing the dialysis bag, 10 mL of PBS (pH=5.5/pH=7.4) was placed into the dialysis bag. The release temperature was set at 37 °C and the rotational speed was set at 200 rpm. 1 mL of dialysate was taken at 1, 3, 6, 12, 24 h and the corresponding preheated PBS was supplemented at the same time. The content of Mn^2+^ was detected by ICP-OES (Agilent 720ES).

### Small Animal *In Vivo* Imaging

Three C57BL/6 male mice were selected for back hair removal. After anesthesia, IR780-labeled (20%) Mn-ZIF-8 MNs were either pressed onto the tumor-bearing skin of each mouse for 2 minutes or injected intratumorally into the subcutaneous tumor., and the fluorescence signals of the back skin of the mice at different time points (1, 2, 6, 12, 24, 48, 72, 96, and 120 hours) were collected using a small animal* in vivo* imaging system (PerkinElmer, USA).

### Cell Culture

The mouse melanoma cell line B16 and the human melanoma cell line A375 were respectively cultured in Roswell Park Memorial Institute (RPMI) 1640 medium (Gibco, Grand Island, NY, USA) and Dulbecco's Modified Eagle's Medium (DMEM, Gibco) containing 10% fetal bovine serum (FBS, ExCell Bio, Shanghai, China) and 1% penicillin-streptomycin (NCM Biotech, Newport, RI, USA) at 37 °C in a 5% CO_2_ atmosphere incubator.

### Cell Viability Assay

The percentage of the viable cells was detected using CCK8 assays. B16 and A375 cells (1 × 10^3^ per well) were seeded into 96-well plates and allowed to attach for 6-8 hours. Then, 100 µL of complete 1640 or DMEM was added to support cell growth. Cells were exposed to additional media containing nanoparticles at different concentrations for 16 h and then subjected to X-ray irradiation at the indicated doses. After culture for another 24 h, 10 µL of the CCK8 (5 mg.mL^-1^) stock solution (GlpBio, China) was added to each well and the plate was incubated for 2 h at 37 °C. The absorbance in each well was measured using a Multifunctional plate reader (TECAN, Männedorf Switzerland) at a wavelength of 450 nm. The relative percentage of the untreated cells was adjusted to represent 100% cell viability, and then the relative viabilities of the treated cells were calculated and plotted as cell survival curves using GraphPad Prism 9.5 software (GraphPad Inc., La Jolla, CA, USA).

### Colony Formation Assay

B16 and A375 cells (2 × 10^3^ per well) were seeded into six-well plates with complete medium and allowed to grow for 6-8 hours. Then, 2 mL of complete 1640 or DMEM was added to support cell growth. The plates were incubated with nanoparticles at the indicated concentrations for 16 h and then with irradiated at the indicated doses of X-rays, followed by further culture for about 10 days. Subsequently, the cells were gently rinsed with phosphate-buffered saline (PBS) three times before being fixed with 4% paraformaldehyde for 15 min at room temperature. After staining with crystal violet (0.1%) for 30 min, the colonies were imaged under a stereomicroscope, analyzed using ImageJ (NIH, Bethesda, MD, USA), then the cell clonal formation rate was plotted as a histogram using GraphPad Prism software.

### *In Vitro* DNA Damage Study

B16 and A375 cells (5 × 10^4^ per well) were seeded into 24-well plates with complete medium and allowed to grow for 6-8 hours. Then, 0.5 mL of complete 1640 or DMEM was added to support cell growth. The plates were incubated with nanoparticles at the indicated concentrations for 16 h, exposed to X-ray irradiation (2Gy), and then further cultured for 1 h. Subsequently, the cells were gently rinsed with PBS three times and before being fixed with 4% paraformaldehyde for 15 min at room temperature. The cells were then washed with PBS three times, incubated with 0.5% TritonX-100 at room temperature for 15 min, washed with PBS twice, submerged in 5% bovine serum albumin (BSA) sealing solution for 1 h, incubated with anti-γ-H_2_AX antibodies (Cell Signaling Technology, USA) at 4 °C overnight, washed with PBS-Tween20 (PBST) three times, and incubated with Alexa Fluor‑conjugated secondary antibodies (Proteintech, Wuhan, China) at room temperature for 1 h. Following three further washes with PBST, the cell nuclei were stained using 4′,6-diamidino-2-phenylindole (DAPI, Beyotime Biotech, Shanghai, China). The stained cells were observed under a confocal microscope (Zeiss, Oberkochen, Germany), photographed, and then analyzed using ImageJ. A histogram of the mean cell fluorescence intensity was plotted using GraphPad Prism software.

### *In Vitro* CRT Exposure, HMGB1 Release, and ATP Secretion Assays

B16 and A375 cells (5 × 10^4^ per well) were seeded into 24-well plates with complete medium and allowed to grow for 6-8 hours. Then, 0.5 mL of complete 1640 or DMEM was added to support cell growth. The plates were incubated with nanoparticles at the indicated concentrations for 16 h, exposed to X-ray irradiation (6 Gy), and then further cultured for 24h or 8h. Subsequently, the cells were gently rinsed with PBS three times and fixed with 4% paraformaldehyde for 15 min at room temperature. Then, the cells washed with PBS three times and stained with anti-HMGB1 or anti-calreticulin antibodies at 4 °C overnight. Next day, the cells were washed with PBST three times, incubated with fluorescently labeled secondary antibodies at room temperature for 1 h, and washed with PBST three times. The cell nuclei were stained using DAPI. The stained cells were observed under a confocal microscope (Zeiss), photographed, and then analyzed using ImageJ. A histogram of the mean cell fluorescence intensity was plotted using GraphPad Prism software.

In a similar experiment, after the cells were treated with RT as above, incubation was continued for 18 h. The cell culture medium was collected, and dying cells in the medium were removed through centrifugation. The supernatants were utilized for quantitative analyses of the ATP content using an ATP assay kit (Beyotime, Shanghai, China). An ATP content histogram was plotted using GraphPad Prism software.

### Detection of Mitochondrial Superoxide Generation *In Vitro*

To evaluate mitochondrial reactive oxygen species (ROS) levels *in vitro*, MitoSOX™ Green (Thermo Fisher Scientific) was used as a mitochondria-targeted fluorescent probe for superoxide detection in B16 melanoma cells. A 1 mM MitoSOX™ Green stock solution was prepared by dissolving the contents of one vial in 10 μL of anhydrous dimethylformamide (DMF). Working solution (1 μM) was freshly prepared by diluting 3 μL of the stock solution in 3 mL Hank's Balanced Salt Solution (HBSS) containing calcium and magnesium. After the indicated treatments (X-ray, ZIF-8, Mn-ZIF-8), the culture medium was aspirated, and cells were washed once with pre-warmed HBSS. Each well was then incubated with 1 mL of 1 μM MitoSOX™ Green working solution for 30 minutes at 37 °C in the dark. After incubation, cells were washed three times with pre-warmed HBSS to remove residual probe. Cells were imaged using a fluorescence microscope equipped with FITC channel (excitation 488 nm, emission 510 nm). Mitochondrial superoxide levels were quantified using ImageJ software based on mean fluorescence intensity in the FITC channel.

### Western Blotting

Levels of proteins related to the STING signaling pathways in B16 and A375 cells incubated with ZIF-8 or Mn-ZIF-8 NPs with or without X-ray were analyzed using western blotting. Total cellular protein extracts were separated by sodium dodecyl sulfate-polyacrylamide gel electrophoresis (SDS-PAGE), transferred onto polyvinylidene difluoride membranes. After blocking with 5% BSA, the membranes were incubated with primary antibody overnight at 4 °C. The membranes were then incubated with secondary antibodies for 60 min. The immunoreactive protein bands were incubated with an ECL kit (Applygen, Beijing, China) and analyzed using an imaging system (Tanon 5200 Multi, Shanghai, China). Greyscale analysis of immunoblot bands was performed using ImageJ software. Antibody information is provided in Supporting Information: Table S1.

### Cytokine Detection

B16 cells (1 × 10^5^ per well) were seeded into 6-well plates with complete medium and allowed to grow for 6-8 hours. Then, 2 mL of complete 1640 medium was added to support cell growth. The plates were incubated with nanoparticles at the indicated concentrations for 16 h, exposed to X-ray irradiation (6 Gy), and then further cultured for 48 h. Cell supernatants were collected and subjected to ELISA for IFN-β detection (Mei Mian Biotechnology Co., Ltd., Jiangsu, China). An IFN-β content histogram was plotted using GraphPad Prism software.

### Mouse Model and Treatment

All animal experiments were approved by the Experimental Animal Protection, Welfare, and Ethics Committee of Nanfang Hospital, Southern Medical University under the protocol number IACUC-LAC-20231022-001. Female 6-week-old C57BL/6 mice were obtained from Guangdong Zhiyuan Biological Pharmaceutical Company and housed under SPF conditions at the Experimental Animal Center of Nanfang Hospital. The animals were maintained under a controlled environment (temperature 20-24 °C, humidity 40-70%, 12 h light/dark cycle) with ad libitum access to standard chow and water. To ensure experimental reproducibility and reduce selection bias, mice were randomly assigned to different treatment groups using a random number generator (GraphPad Prism 9). Tumor-bearing mice were included in the study only after the subcutaneous tumor volume reached approximately 80-100 mm³ to ensure baseline homogeneity. All procedures adhered to the National Institutes of Health Guide for the Care and Use of Laboratory Animals, which outlines standards for humane endpoints, anesthesia, and euthanasia protocols. B16 cells (5 × 10^5^) were injected subcutaneously into the right lateral thigh area of the C57/BL6 mice. B16 cells (3 × 10^5^) were subcutaneously injected in the left side of the mice on the second day. On day 6, when the right tumor reached approximately 100 mm^3^, the animals were randomly assigned to the control and different treatment groups. On the seventh day after tumor implantation, the MNs patches were applied to the tumor site. On day 8, all mice were anesthetized by intraperitoneal (i.p.) injection of pentobarbital sodium (20 mg/kg), and the tumors were irradiated once with X-ray (12 Gy) using the Small Animal Radiation Research Platform of Southern Hospital radiotherapy department (512 cGy/min, 6-MeV-ray beam; Siemens, Munich, Germany). An anti-mouse PD-1 monoclonal antibody (mAb) (Bio X Cell, Lebanon, NH, USA) was administered intraperitoneally (200 μg per mouse) on days 8, 10 and 12. The tumor volumes and body weights were monitored every 2-3 days from tumor implantation until the mice were euthanized. The tumor volume was calculated using the following formula: tumor volume (mm^3^) = width^2^ (mm^2^) × length (mm) × 0.5. After 16 or 18 days, the mice were sacrificed, and the tumors and spleens were weighed. Animals were euthanized when they showed signs of imperfect health or when the size of tumors exceeded 2000 mm^3^.

### *In Vivo* Safety Evaluation

The *in vivo* toxicity of ZIF-8 or Mn-ZIF-8 MNs under irradiation was evaluated in healthy C57BL/6 mice (6 weeks old). The grouping and other parameters were consistent with the *in vivo* antitumor efficacy tests. The body weights of the mice were recorded until day 18. Blood samples were collected on day 18, and blood cells counts were determined. Then, the blood biochemical values, including the levels of serum alanine transaminase (ALT), aspartate transaminase (AST), total bilirubin (TBIL), blood urea nitrogen (BUN), uric acid (UA), and creatinine (CREA), were analyzed to investigate potential hepatic and renal toxicity. On the day 18, the major organs (heart, liver, spleen, lung, and kidney) were removed and analyzed using H&E staining.

### Flow Cytometry

The therapeutic impact of ZIF-8 or Mn-ZIF-8 MNs under radiation was assessed. In a unilateral tumor-bearing mouse model, at day 18 post-implantation, the animals were euthanized, and samples of the tumor, spleen, and ipsilateral inguinal lymph nodes were harvested. For the bilateral tumor-bearing model, on day 16 post‑implantation, the animals were euthanized, and both the primary and metastatic tumors were collected. Subsequently, single-cell suspensions from lymph nodes, spleen, and tumors were prepared using an enzyme cocktail (comprising neutral protease, collagenase type II, and hyaluronidase) (BD Biosciences, San Jose, CA, USA). The cells from the inguinal lymph nodes were stained with anti-CD11c-PerCP-Cy5.5, anti-CD86-PE-Cy7, and anti-CD80-BV421 antibodies) for the DC maturation study. Tumor cells were stained with anti-CD3-PerCP-Cy5.5, anti-CD4-BV510, anti-CD8-FITC, anti-anti-forkhead box P3 (FoxP3)-BV421, and anti-CD45-PE antibodies and then analyzed using flow cytometry. Antibody information is provided in Supporting Information: Table S2. The gating strategy is provided in Figure S34.

### Histopathology and Immunohistochemistry

Biopsied tissue samples were subjected to H&E staining to facilitate histological evaluation. For the immunohistochemical procedures, both primary and metastatic lesions were extracted from rodents across various treatment arms and preserved in formaldehyde solution (4%, w/v). Post-paraffin embedding, the tumor tissues deparaffinized and rehydrated, preceding heat induced antigen retrieval. The tumor tissue sections were stained with primary antibodies (Abcam, Cambridge, MA, USA) comprising anti-Ki67, anti-CD4, anti-CD8, FoxP3 and GZMB and then reacted with horseradish peroxidase (HRP)-labeled secondary antibodies (DAKO, Glostrup, Denmark). Images were captured using an automatic slide scanner after visualization using a 3,3′-Diaminobenzidine (DAB) substrate kit (DAKO). Antibody information is provided in Supporting Information: Table S3.

### Statistical Analysis

All data are displayed as mean values ± standard deviation (SD). The significance of the statistical differences among the groups was determined using one-way ANOVA and Tukey's multiple comparisons test. The threshold for statistical significance was as follows: *, p values < 0.05; **, p values < 0.01; ***, p values < 0.001; and ****, p values < 0.0001. All statistical analyses were carried out with GraphPad Prism 9.5 software).

## Supplementary Material

Supplementary figures and tables.

## Abbreviations

RT: radiotherapy
cGAS: cyclic GMP-AMP synthase
STING: stimulator of interferon response cGAMP interactor
ICD: immunogenic cell death
MNs: microneedles
ZIF-8: zeolite imidazolate frame-8
DCs: dendritic cells
TME: tumor microenvironment
ICIs: immune checkpoint inhibitors
ECM: extracellular matrix
ICB: immune checkpoint blockade
IFN: type I interferon
PD-1: anti-programmed cell death 1
αPD-1: anti-programmed cell death 1 antibodies
MOF: metal-organic framework
NPs: nanoparticles
IRF3: interferon regulatory factor 3
IFN-β: interferon beta
TEM: transmission electron microscopy
XRD: X-ray diffraction
XPS: X-ray photoelectron spectroscopy
IR: irradiation
CCK8: cell couniting kit-8
DAMPs: damage-associated molecular patterns
CRT: calreticulin
HMGB1: high mobility group box 1
H&E: hematoxylin and eosin
IHC: immunohistochemistry
Ki-67: proliferation Ki-67
Tregs: regulatory T cells
GZMB: granzyme B
HA: hyaluronic acid
PDMS: polydimethylsiloxane
PVP K90: polyvinylpyrrolidone
RPMI: Roswell Park Memorial Institute
DMEM: Dulbecco's Modified Eagle's Medium
FBS: fetal bovine serum
PBS: phosphate-buffered saline
BSA: bovine serum albumin
PBST: PBS-Tween20
ROS: reactive oxygen species
DMF: dimethylformamide
HBSS: Hank's Balanced Salt Solution
SDS-PAGE: sodium dodecyl sulfate-polyacrylamide gel electrophoresis
i.p.: intraperitoneal
mAb: monoclonal antibody
ALT: alanine transaminase
AST: aspartate transaminase
TBIL: total bilirubin
BUN: blood urea nitrogen
UA: uric acid
CREA: creatinine
FoxP3: anti-anti-forkhead box P3
HRP: horseradish peroxidase
DAB: 3,3′-Diaminobenzidine
DAKO: 3,3′-Diaminobenzidine substrate kit
SD: standard deviation
γ-H_2_AX: gamma-histone H_2_AX
ATP: adenosine triphosphate
