# Supplementary material for: X-ray-responsive dissolving microneedles mediate STING pathway activation to potentiate cutaneous melanoma radio-immunotherapy
Source: Theranostics. 2025 Jun 9;15(14):6919–37. doi: 10.7150/thno.110841 (PMC12203811; doi:10.7150/thno.110841)
Supplement: Supplementary file 1 — Supplementary figures and tables. [file thnov15p6919s1.pdf]

Supporting Information

X-Ray-Responsive Dissolving Microneedles Mediate STING Pathway Activation to Potentiate Melanoma Radio-Immunotherapy

Wen Hu, Xiaohong Hong, Xinyu Zhang, Hongfan Chen, Xin Wen, Feng Lin, Jingwen Liu, Chenfenglin Yang, Binglin Cheng, Hanrui Zhu, Moting Zhang, Ruzhen Chen, Tingting Peng, and Xinran Tang**

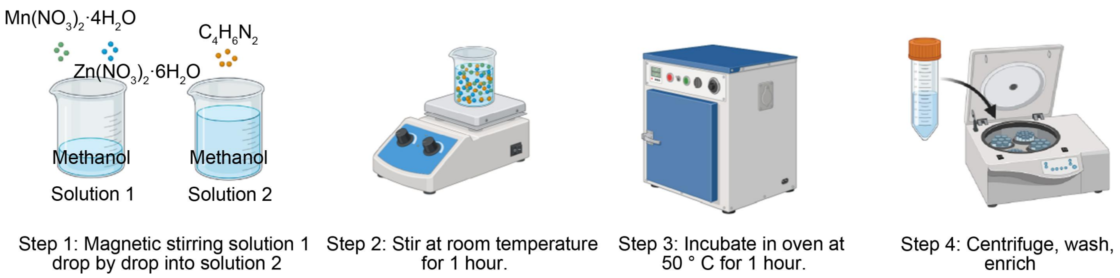

Figure S1. Schematic diagram of the synthesis of Mn-ZIF-8 nanoparticles.

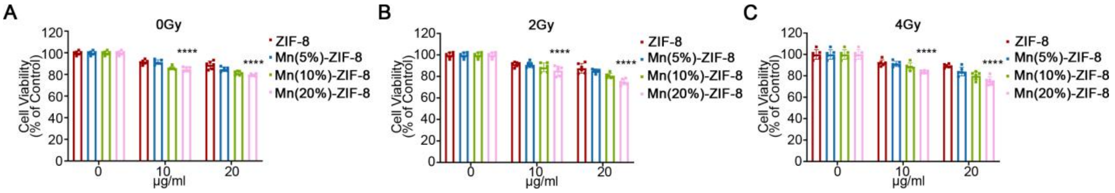

Figure S2. Effect of Mn-ZIF-8 at different concentrations and its components in combination with radiotherapy (0, 2, 4 Gy) on the viability of B16 cells (*n* = 6 per group). The data are presented as the mean ± SD. ****$P < 0.0001$.

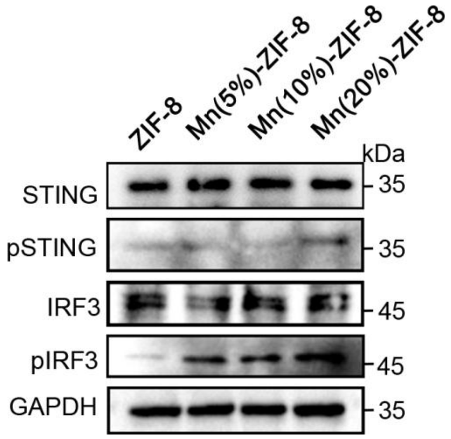

Figure S3. WB analysis of STING pathway protein activation in B16 cells treated with 20 μg/ml ZIF-8 and Mn-ZIF-8 at different Mn^{2+} doping ratios, in combination with radiotherapy (6 Gy).

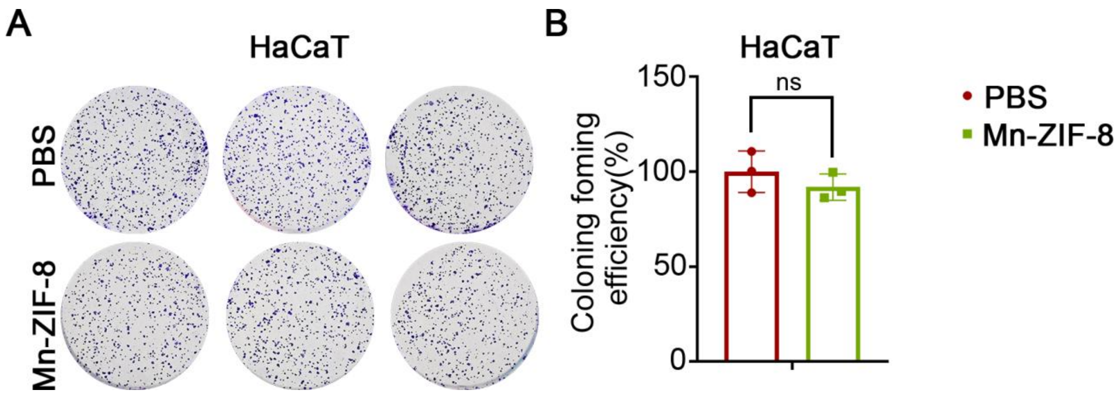

Figure S4. Effect of PBS and Mn-ZIF-8 on the proliferation of HaCaT cells, as assessed using a colony formation assay (n = 3 per group). The data are presented as the mean ± SD. ns $P > 0.05$.

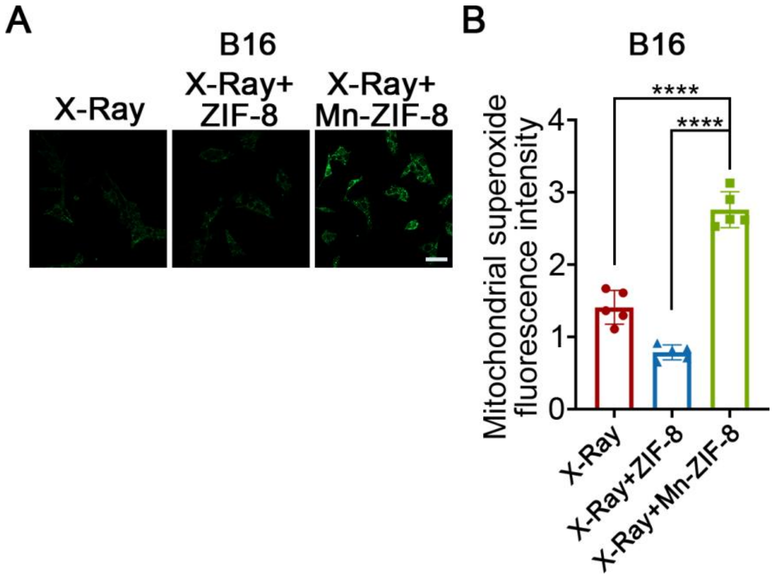

Figure S5. Quantification of mitochondrial superoxide fluorescence intensity and representative fluorescence images of melanoma cells subjected to various treatments (n = 5 per group). The data are presented as the mean ± SD. ****P < 0.0001.

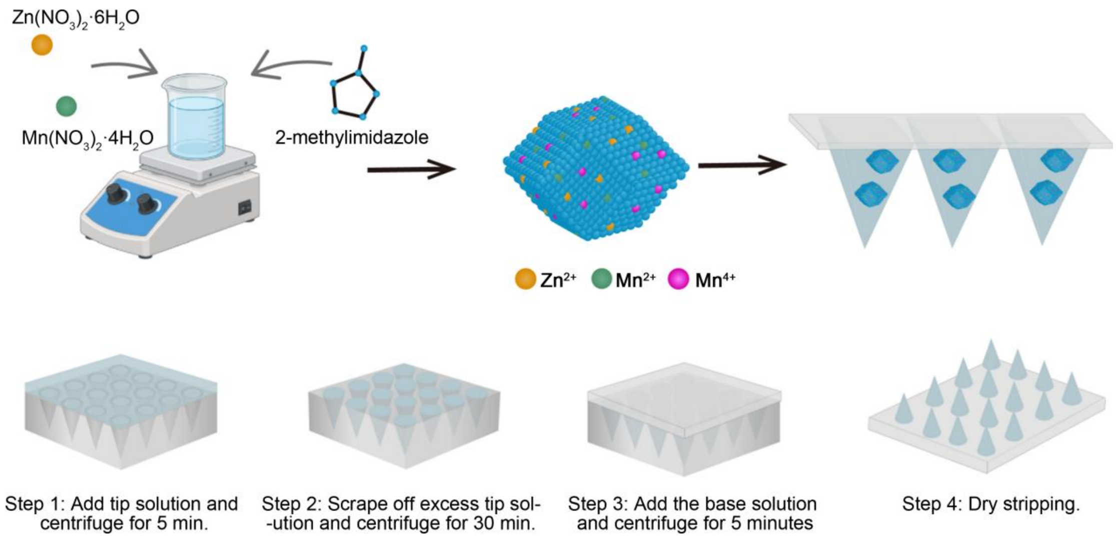

Figure S6. Schematic diagram of the synthesis of Mn-ZIF-8 MNs.

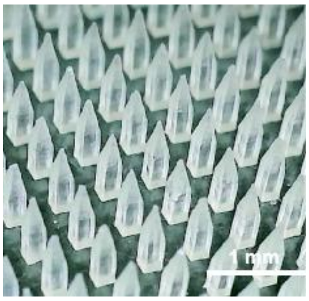

Figure S7. Photograph of ZIF-8 MNs array.

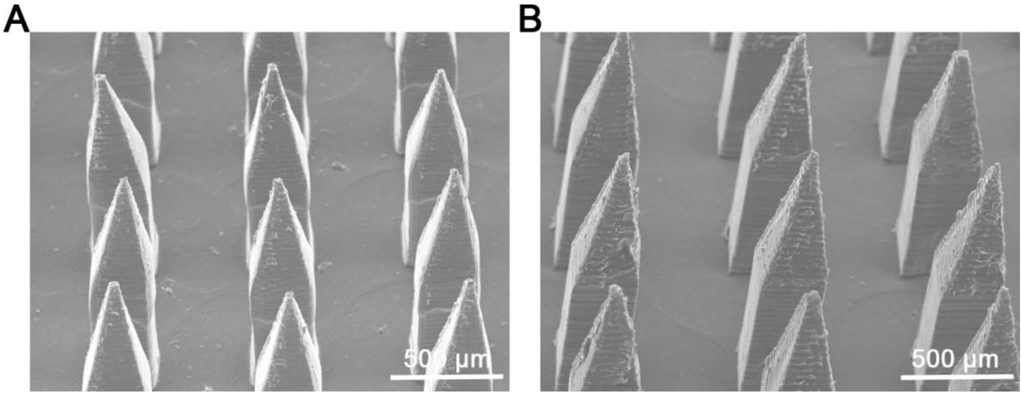

Figure S8. SEM images of Mn(20%)-ZIF-8 MNs (left) and ZIF-8 MNs (right).

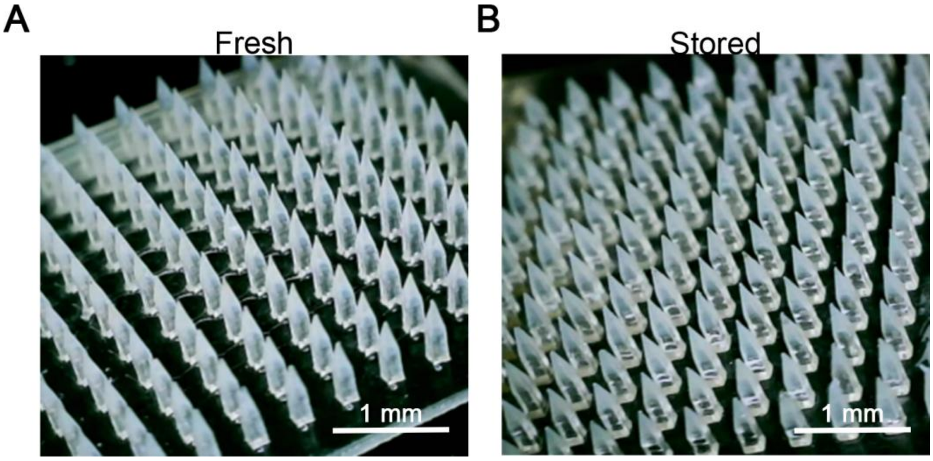

Figure S9. Photograph of the fresh (A) and stored (B) Mn-ZIF-8 MNs array.

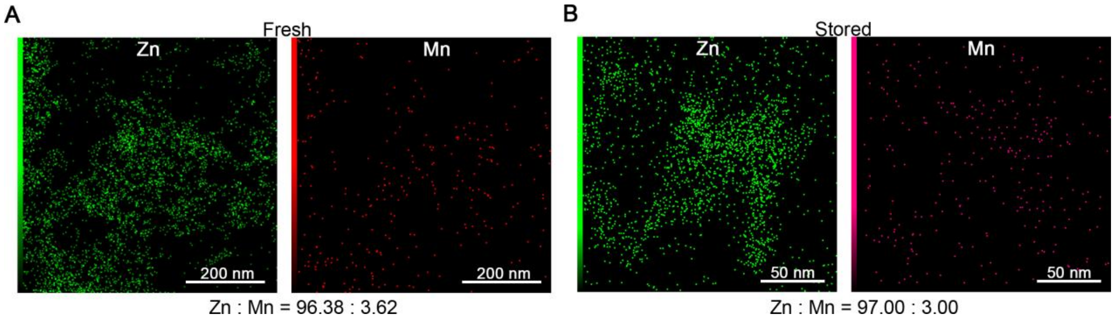

Figure S10. Elemental mapping images and the elemental ratios of Zn and Mn remained consistent between the fresh (A) and stored (B) Mn-ZIF-8 MNs.

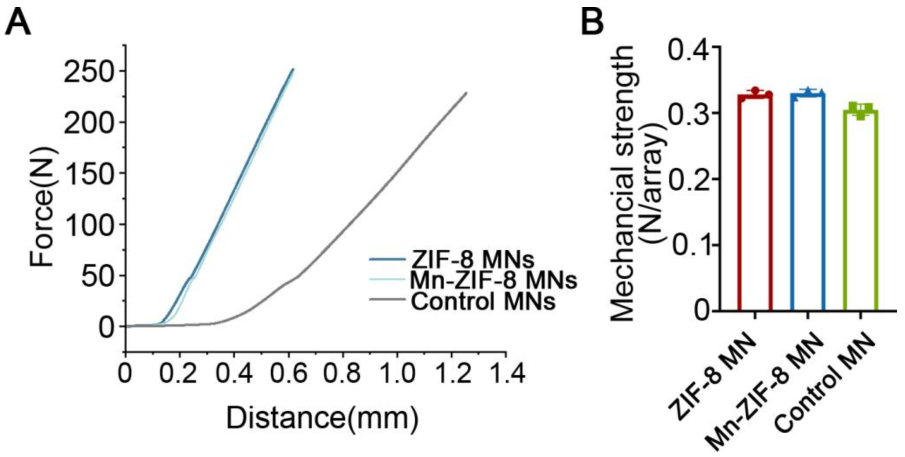

Figure S11. (A) The force-displacement curves of ZIF-8, Mn(20%)-ZIF-8 and Control MNs. (B) Mechanical strength of the force per individual needle (N per needle) (n = 3 per group). The data are presented as the mean ± SD.

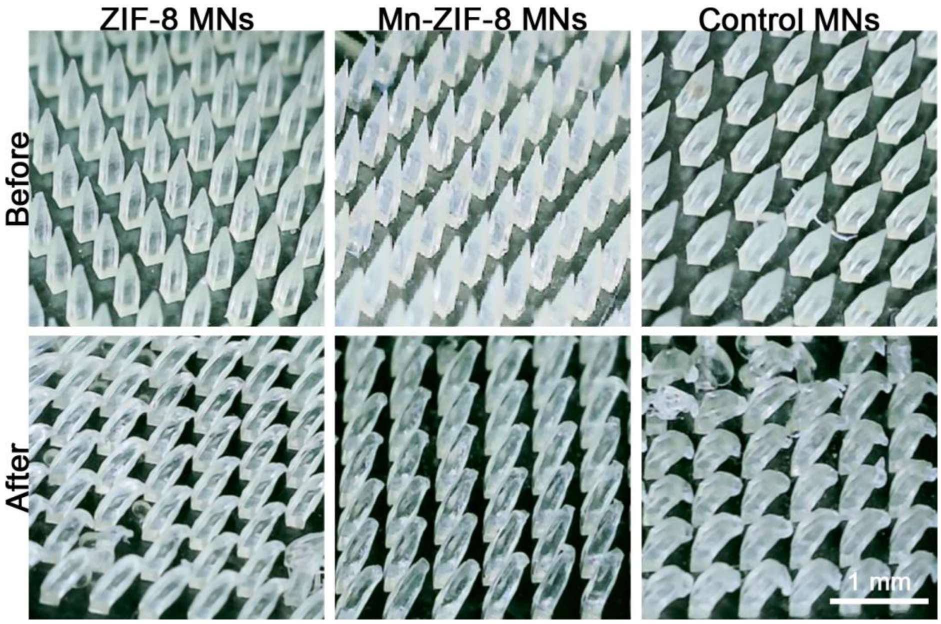

Figure S12. The morphology of the microneedles both before and after mechanical testing.

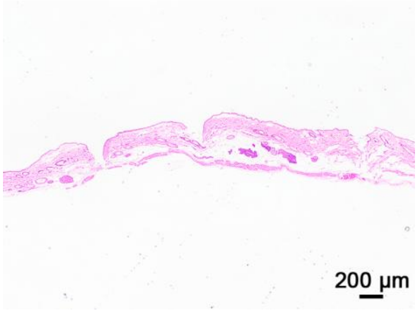

Figure S13. H&E staining of the rat skin punctured with ZIF-8 MNs.

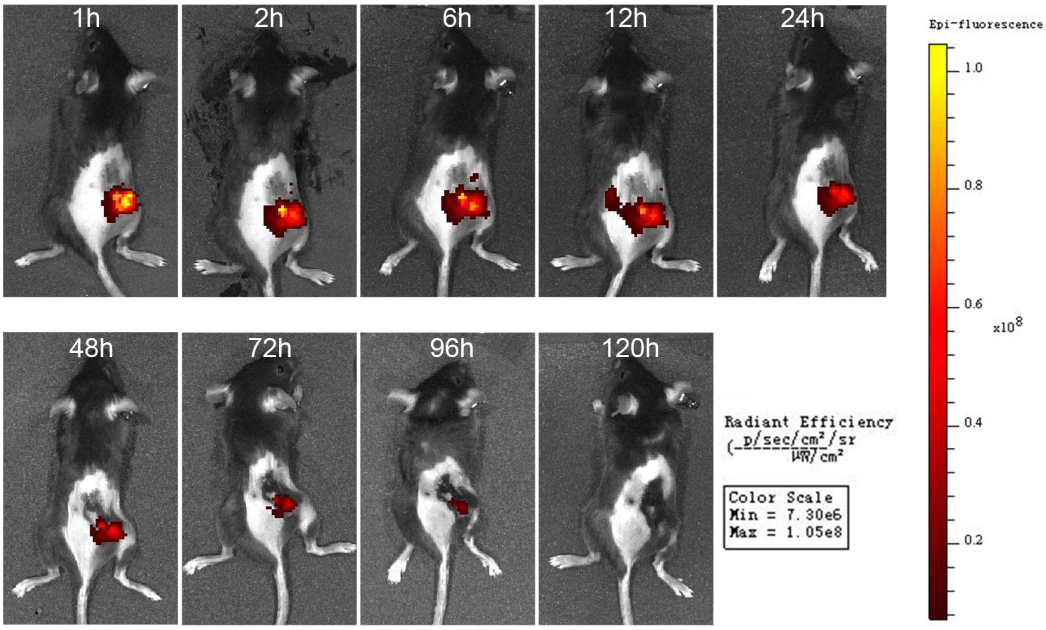

Figure S14. *In vivo* imaging of the melanoma-bearing mice after administration of Mn-ZIF-8 MNs for the indicated time.

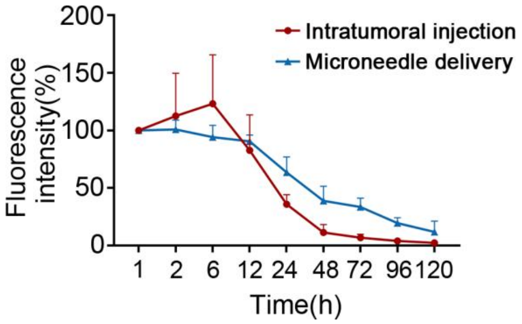

Figure S15. The average fluorescence intensity of the local skin over time. The data are presented as the mean ± SD; *n* = 3 per group.

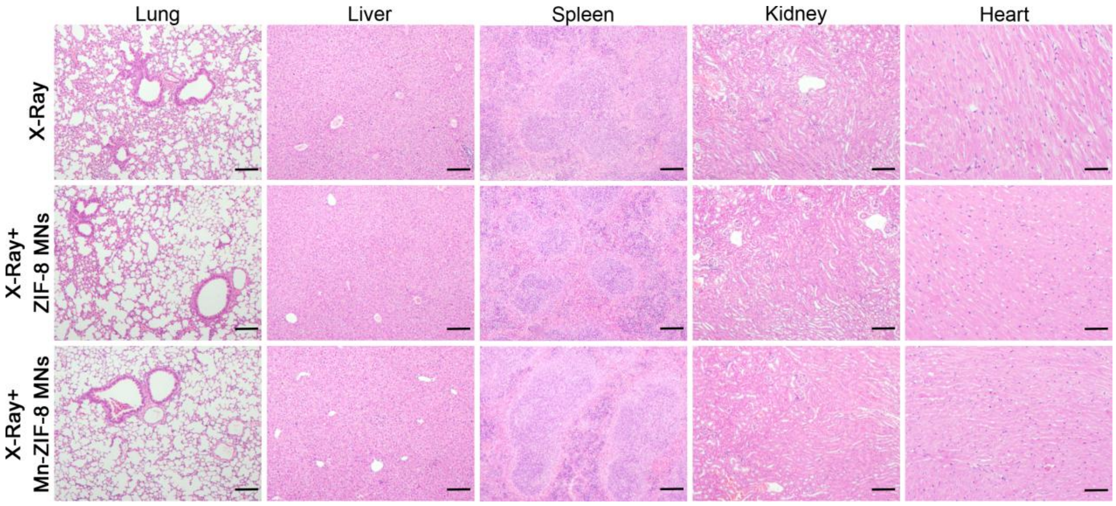

Figure S16. H&E staining of the major organs harvested 18 days after treatments. Scale bar = 100 μm.

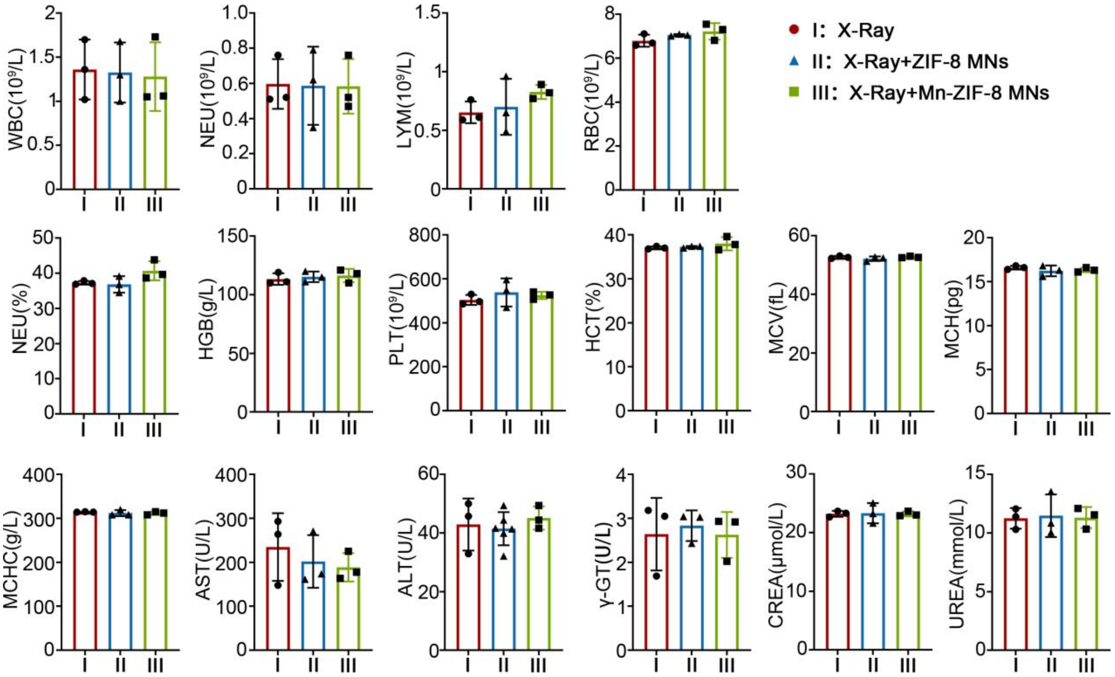

Figure S17. The whole blood panel analysis and blood biochemistry data of tumor-bearing mice after treatments. The data are presented as the mean ± SD; n = 3 per group.

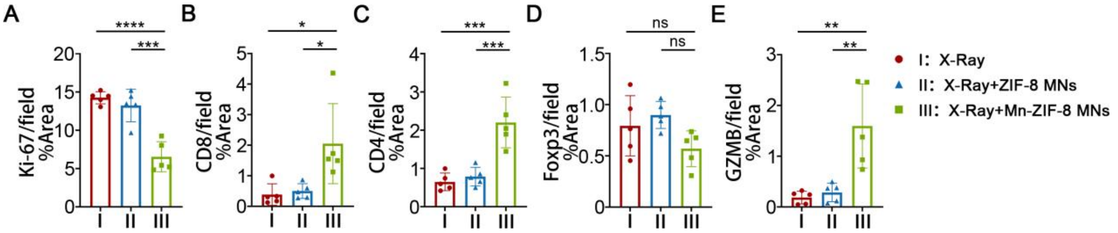

Figure S18. Quantitative analysis of Ki67, CD8, CD4, Foxp3 and GZMB expression after the indicated treatments in the unilateral tumor mouse model (n = 5 per group). The data are presented as the mean ± SD. The data are presented as the mean ± SD. ns $P > 0.05$, *$P < 0.05$, ***$P < 0.001$ and ****$P < 0.0001$.

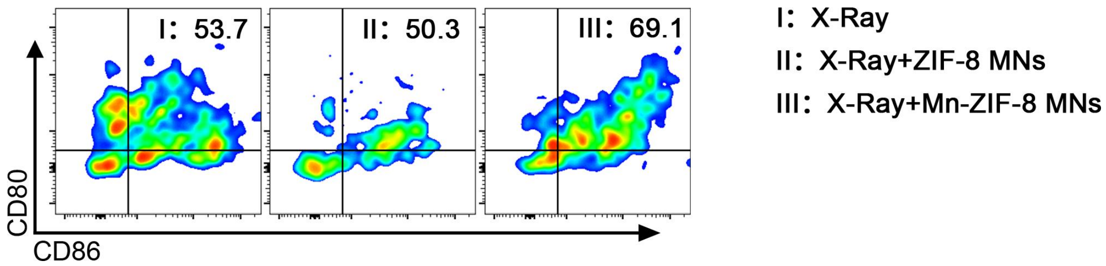

Figure S19. The representative flow cytometric plots of matured DCs (CD80$^+$ CD86$^+$ in CD11c$^+$ cells) in inguinal lymph nodes adjacent to tumors after treatments. Data related to Figure 5J.

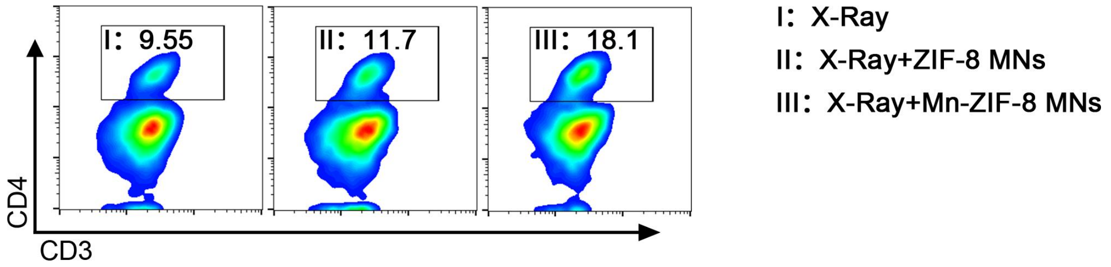

Figure S20. The representative flow cytometric plots of CD4$^+$ T in tumors after treatments. Data related to Figure 5K.

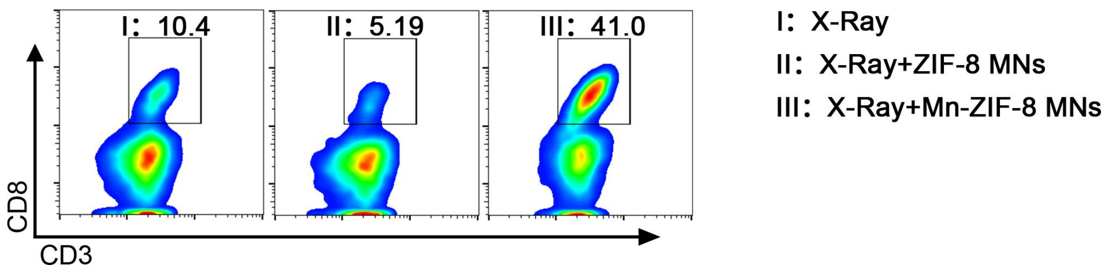

Figure S21. The representative flow cytometric plots of CD8[+] T in tumors after treatments. Data related to Figure 5L.

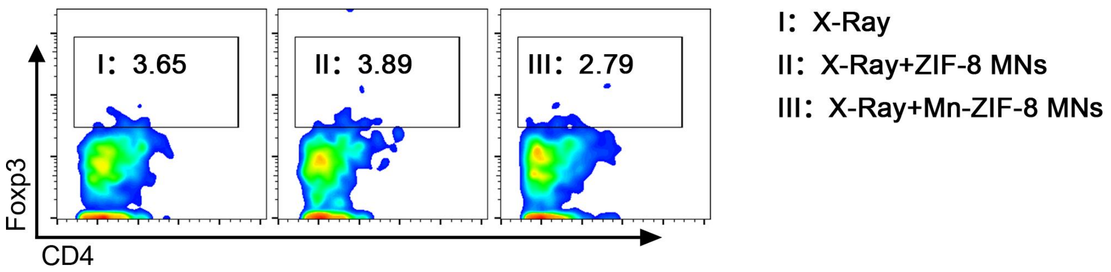

Figure S22. The representative flow cytometric plots of Treg in tumors after treatments. Data related to Figure 5M.

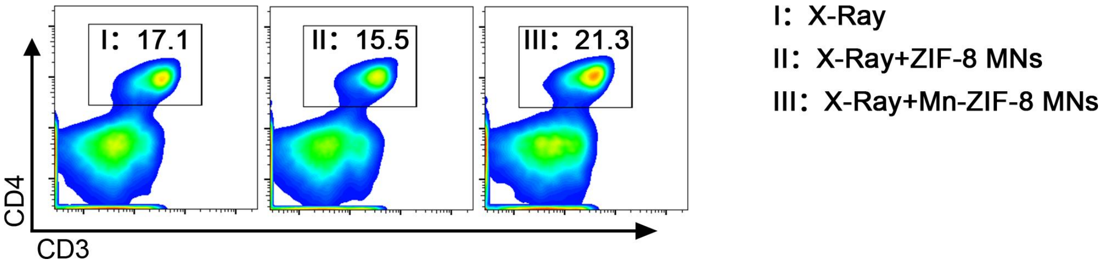

Figure S23. The representative flow cytometric plots of CD4^{+} T in spleen after treatments. Data Related to Figure 5N.

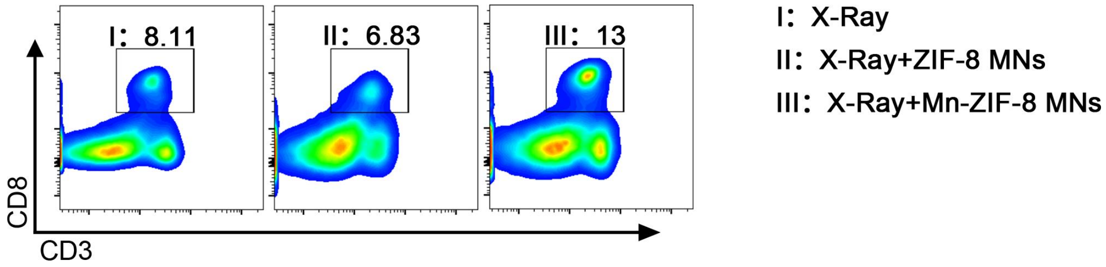

Figure S24. The representative flow cytometric plots of $CD8^+$ T in spleen after treatments. Data related to Figure 5O.

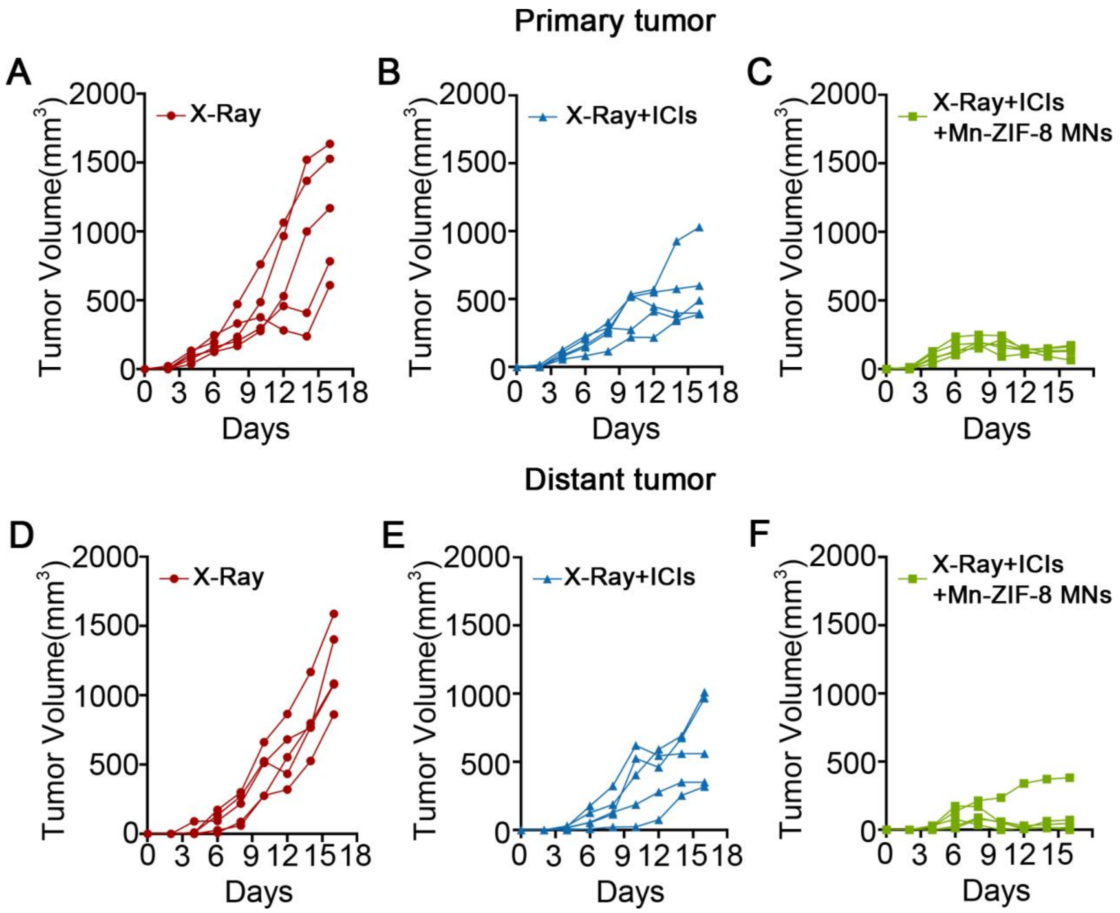

Figure S25. Individual tumor growth curves of mice after different treatments. Data related to Figure 6E, F.

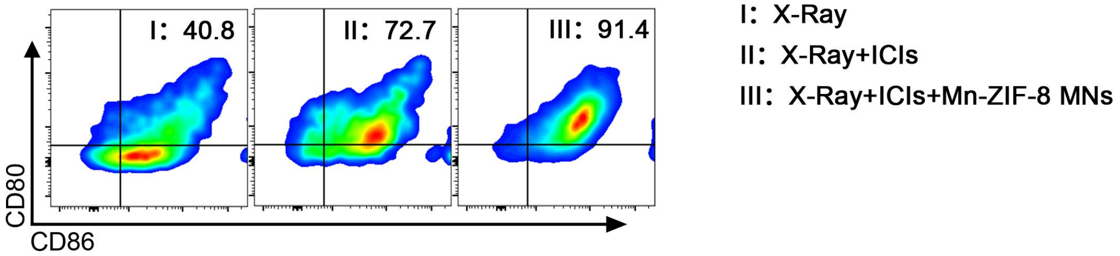

Figure S26. The representative flow cytometric plots of matured DCs ($CD80^+$ $CD86^+$ in $CD11c^+$ cells) in inguinal lymph nodes adjacent to primary tumors after treatments. Data related to Figure 6G.

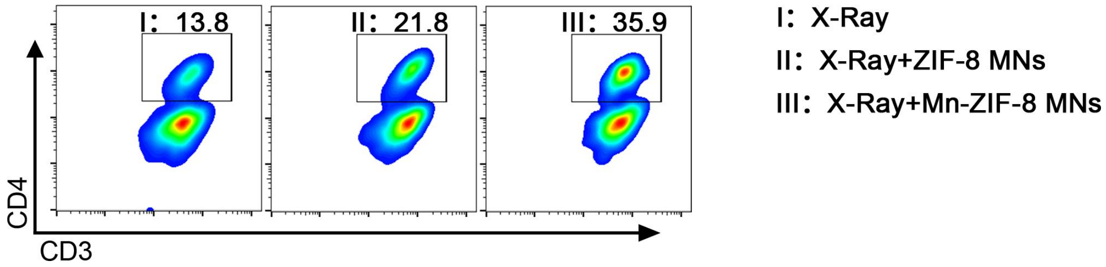

Figure S27. The representative flow cytometric plots of CD4^{+} T in primary tumors after treatments. Data related to Figure 6H.

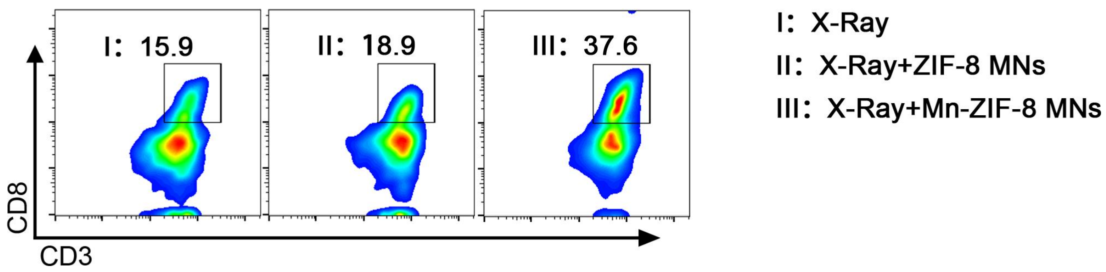

Figure S28. The representative flow cytometric plots of $CD8^+$ T in primary tumors after treatments. Data related to Figure 6H.

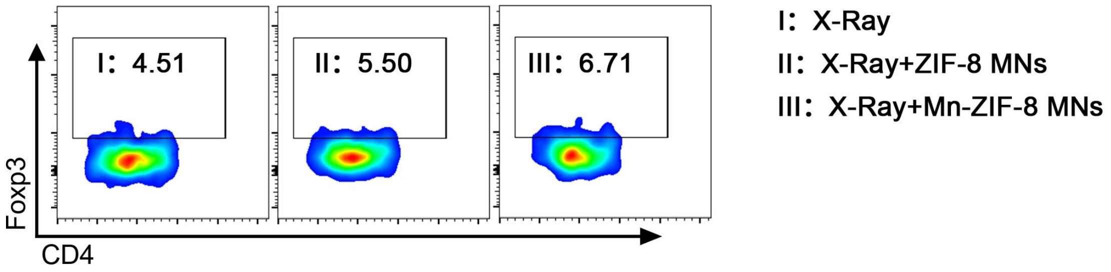

Figure S29. The representative flow cytometric plots of Treg in primary tumors after treatments. Data related to Figure 6H.

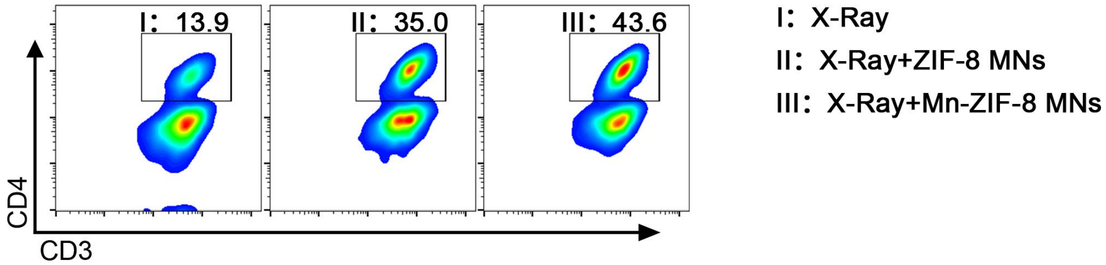

Figure S30. The representative flow cytometric plots of $CD4^+$ T in distant tumors after treatments. Data related to Figure 6I.

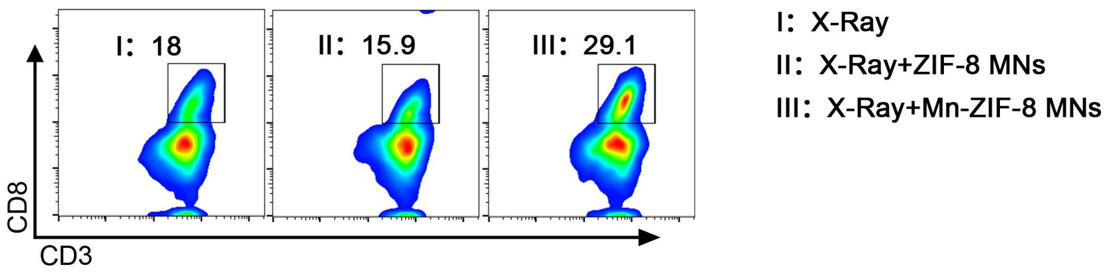

Figure S31. The representative flow cytometric plots of $CD8^{+}$ T in distant tumors after treatments. Data related to Figure 6I.

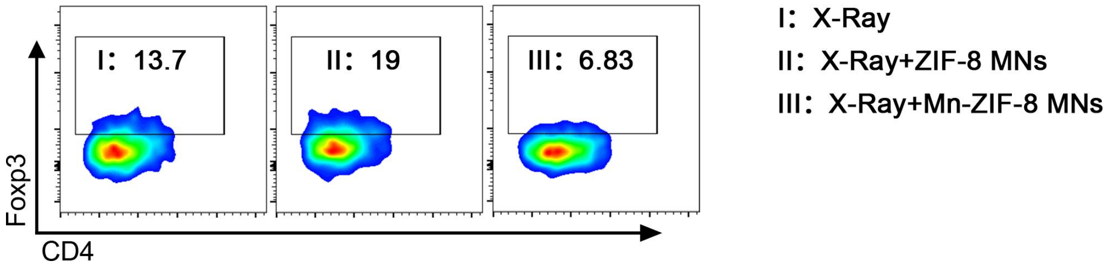

Figure S32. The representative flow cytometric plots of Treg in distant tumors after treatments. Data related to Figure 6I.

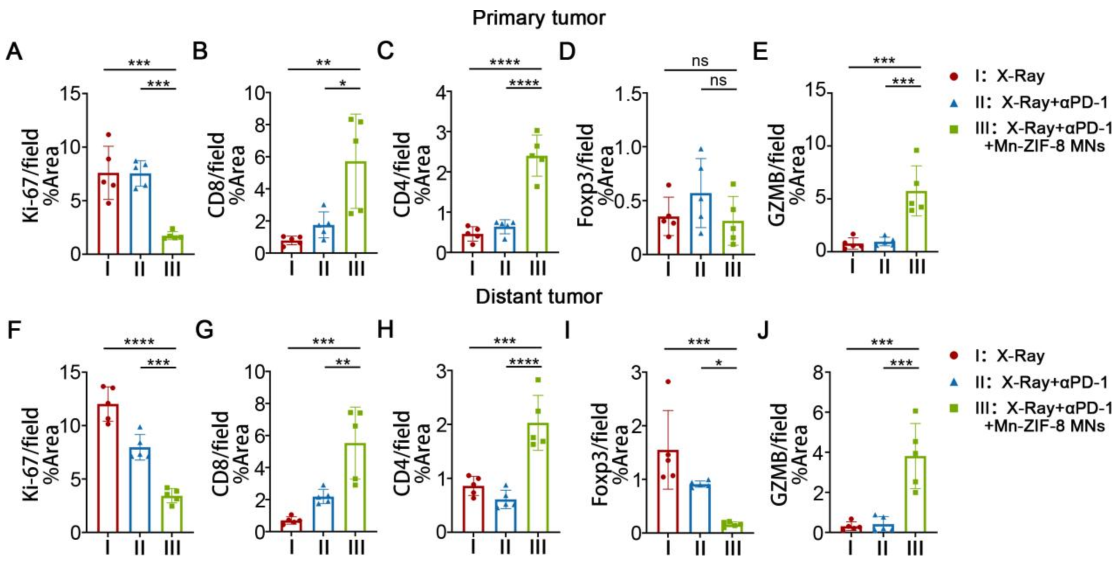

Figure S33. Quantitative analysis of Ki67, CD8, CD4, Foxp3 and GZMB expression after the indicated treatments in the bilateral tumor mouse model (n = 5 per group). The data are presented as the mean ± SD. ns $P > 0.05$, *$P < 0.05$, **$P < 0.01$, ***$P < 0.001$ and ****$P < 0.0001$.

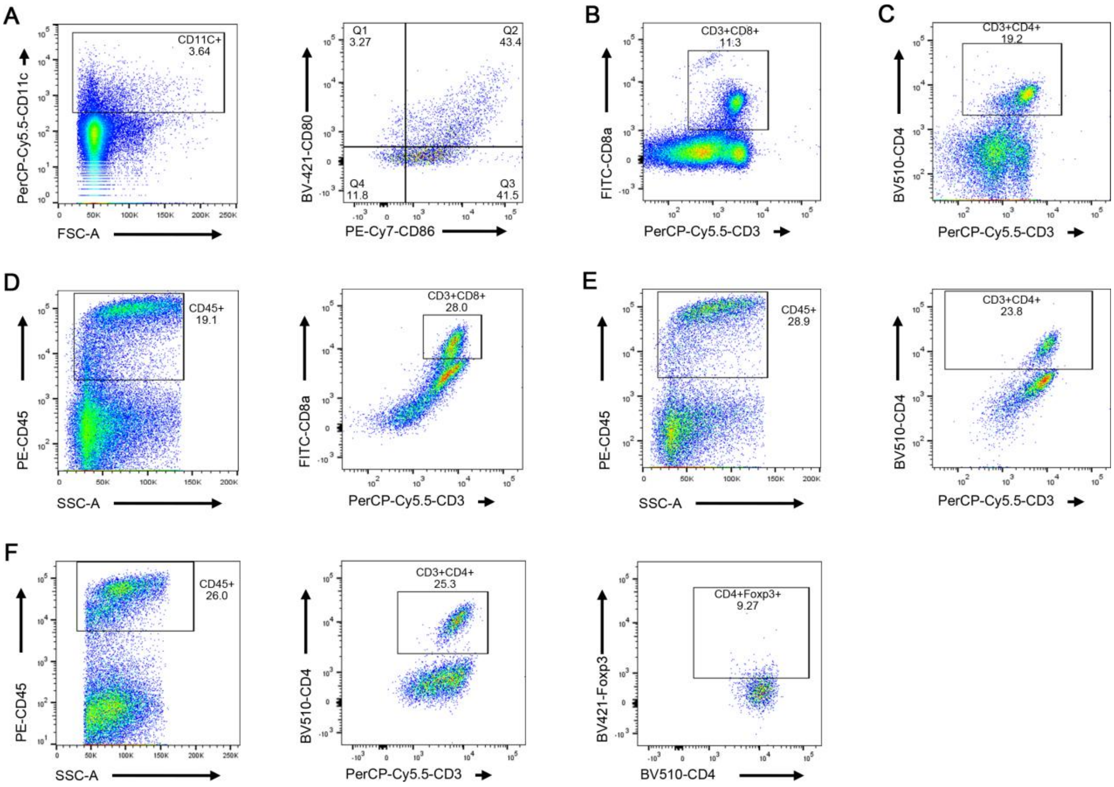

Figure S34. Gating strategy for flow cytometric analysis.

A, Gating strategy for the flow cytometric analysis in Fig. 5J, Fig. 6G and Supplementary Fig. S26.

B, Gating strategy for the flow cytometric analysis in Fig. 5O and Supplementary Fig. S24.

C, Gating strategy for the flow cytometric analysis in Fig. 5N and Supplementary Fig. S23.

D, Gating strategy for the flow cytometric analysis in Fig. 5L, 6H, 6I and Supplementary Fig. S21, S28, S31.

E, Gating strategy for the flow cytometric analysis in Fig. 5K, 6H, 6I and Supplementary Fig. S20, S27, S30.

F, Gating strategy for the flow cytometric analysis in Fig. 5M, 6H, 6I and Supplementary Fig. S22, S29, S32.

Table S1. List of antibodies used for Western blotting (WB).

	Supplier	Catalogue No.	Host species	Species activity	Application
Primary antibodies					
anti-STING	CST	13647	Rabbit	Hu, Mo	WB
anti-p-STING	CST	72971	Rabbit	Mo	WB
anti-IRF3	Proteintech	66670-1-IG	Mouse	Hu, Mo	WB
anti-p-IRF3	CST	29047S	Rabbit	Hu, Mo	WB
anti-GAPDH	Proteintech	10494-1-AP	Rabbit	Hu, Mo	WB
Secondary antibodies					
anti-rabbit	CST	7074S	Goat	Rabbit	WB
anti-mouse	CST	7074S	Horse	Mouse	WB

Table S2. List of antibodies used for flow cytometric analysis.

	Supplier	Catalogue No.	Host species	Species activity	Application
PerCP-Cy5.5-anti-CD11c	Biolegend	560584	HL3	Mouse	FC
PE-Cy7-anti-CD86	Biolegend	560582	GL1	Mouse	FC
BV421-anti-CD80	Biolegend	562611	16-10A1	Mouse	FC
PerCP-Cy5.5-anti-CD3	Biolegend	551163	145-2C11	Mouse	FC
BV510-anti-CD4	Biolegend	563106	RM4-5	Mouse	FC
FITC-anti-CD8a	Biolegend	553030	53-6.7	Mouse	FC
BV421-anti-Foxp3	Biolegend	562996	MF23	Mouse	FC
PE-anti-CD45	Biolegend	553081	30-F11	Mouse	FC

Table S3. List of antibodies used for immunofluorescence (IF) and immunohistochemical (IHC) staining.

	Supplier	Catalogue No.	Host species	Species activity	Application
Primary antibodies					
anti-H_2AX	CST	9718	Rabbit	Hu, Mo	IF
anti-CRT	Sabbiotech	48841 SAB	Rabbit	Hu, Mo	IF
anti-HMGB1	Sabbiotech	486066 SAB	Rabbit	Hu, Mo	IF
anti-Ki-67	Servicebio	GB111141	Rabbit	Mo	IHC
anti-CD8	Servicebio	GB15068	Rabbit	Mo	IHC
anti-CD4	Servicebio	GB15064	Rabbit	Mo	IHC
anti-Foxp3	Servicebio	GB112325	Rabbit	Hu, Mo	IHC
Secondary antibodies					
Alexa Fluor 488	Proteintech	SA00013-2	Goat	Rabbit	IF
Alexa Fluor 594	Proteintech	SA00013-4	Goat	Rabbit	IF

Table S4. Compositions of ZIF-8 and Mn-ZIF-8.

Nanoparticles	$Mn(NO_3)_2 \cdot 4H_2O$ (mM)	$Zn(NO_3)_2 \cdot 6H_2O$ (mM)
ZIF-8	0	2.5
5% Mn-ZIF-8	0.125	2.375
10% Mn-ZIF-8	0.25	2.25
20% Mn-ZIF-8	0.5	2
